# Influence of the Dabcyl group on the cellular uptake of cationic peptides: short oligoarginines as efficient cell-penetrating peptides

**DOI:** 10.1007/s00726-021-03003-w

**Published:** 2021-05-25

**Authors:** Ildikó Szabó, Françoise Illien, Levente E. Dókus, Mo’ath Yousef, Zsuzsa Baranyai, Szilvia Bősze, Shoko Ise, Kenichi Kawano, Sandrine Sagan, Shiroh Futaki, Ferenc Hudecz, Zoltán Bánóczi

**Affiliations:** 1 MTA-ELTE Research Group of Peptide Chemistry, Eötvös Loránd Research Network (ELKH), Eötvös L. University, Budapest, Hungary; 2 Sorbonne Université, École normale supérieure, PSL University, CNRS, Laboratoire des biomolécules, LBM, 75005 Paris, France; 3 Department of Organic Chemistry, Eötvös L. University, Pázmány P. Setany 1/A, Budapest, 1117 Hungary; 4 Institute for Chemical Research, Kyoto University, Uji, Kyoto 611-0011 Japan

**Keywords:** Cell-penetrating peptides, Oligoarginine, Cationic cell-penetrating peptides, Drug conjugates, Antitumor activity

## Abstract

Cell-penetrating peptides (CPPs) are promising delivery vehicles. These short peptides can transport wide range of cargos into cells, although their usage has often limitations. One of them is the endosomatic internalisation and thus the vesicular entrapment. Modifications which increases the direct delivery into the cytosol is highly researched area. Among the oligoarginines the longer ones (*n* > 6) show efficient internalisation and they are well-known members of CPPs. Herein, we describe the modification of tetra- and hexaarginine with (4–((4–(dimethylamino)phenyl)azo)benzoyl) (Dabcyl) group. This chromophore, which is often used in FRET system increased the internalisation of both peptides, and its effect was more outstanding in case of hexaarginine. The modified hexaarginine may enter into cells more effectively than octaarginine, and showed diffuse distribution besides vesicular transport already at low concentration. The attachment of Dabcyl group not only increases the cellular uptake of the cell-penetrating peptides but it may affect the mechanism of their internalisation. Their conjugates with antitumor drugs were studied on different cells and showed antitumor activity.

**Supplementary Information:**

The online version contains supplementary material available at 10.1007/s00726-021-03003-w.

## Introduction

Several short peptides with various sequences were described as cell-penetrating peptides (CPPs). These compounds can internalise into different cells and deliver cargos attached to them chemically or physically into the cytosol (Hudecz et al. 2005). Although very different sequences were found we know little about the mechanism of internalisation and about the factors which affect it. Thus the prediction of internalisation ability or the design of this kind of peptides is a challenge (Kalafatovic and Giralt 2017). Many of the CPPs are positively charged and some of them are amphipathic. Based on structure–activity studies it seems that these two properties are very important for the penetration (Kauffman et al. 2015). The sequence of the first described CPPs, Tat (Green and Loewenstein 1988) and penetratin (Derossi et al. 1994) induced the early studies to determine the role of positively charged amino acids in the cellular uptake (Mitchell et al. 2000; Futaki et al. 2001). It turned out that guanidine group and the length of oligopeptides play crucial role in the internalisation. Oligoarginine with 8–12 residues were the most effective ones. Peptoid transporters were also examined in which the position of guanidine groups was changed (Wender et al. 2000). At the beginning, data about the mechanisms of internalisation were very controversial. Nowadays, two main pathways of the cellular uptake were identified, namely direct translocation and endocytosis. The extent of the internalisation shows huge difference among the CPPs. The direct internalisation is preferred because in this way the conjugates may avoid the vesicle encapsulation, and the cargo can reach directly its intracellular target. To increase its value several modifications were examined (e.g. cyclisation (Lättig-Tünnemann et al. 2011; Mandal et al. 2011; Qian et al. 2016; Amoura et al. 2019), attachment of fatty acid (Pham et al. 2004; Lee and Tung 2010; Katayama et al. 2011; Swiecicki et al. 2015)).

Dabcyl group (4–((4–(dimethylamino)phenyl)azo)benzoyl) is a well-known dark quencher in different FRET systems (Tompa et al. 2004). It was also used to develop calpain-specific CPP substrate (Bánóczi et al. 2008a). It turned out that internalisation of FRET substrate peptide is significantly higher than those of substrate without Dabcyl group (unpublished data). It was also demonstrated, the presence of Dabcyl group with high density on the nanoparticle’s surface can be effectively increase its cellular uptake ability (Roloff et al. 2018).

Octaarginine as a well-known CPP is frequently used to deliver wide range of cargos (e.g. small drug molecules (Miklán et al. 2007, 2011; Bánóczi et al. 2008b, 2010, 2018), and peptides into cells (Bánoczi et al. 2007; Bánóczi et al. 2008a; Szabó et al. 2016). In some cases it is failed to deliver efficiently the cargo into the cells, thus their biological activity was poor; for example in case of methotrexate and its pentaglutamylated derivative (Szabó et al. 2016).

In this paper, we describe our findings of the effect of Dabcyl group on the internalisation of short oligoarginines. The tetra- and hexaarginine were modified with Dabcyl group at their N-terminus. This modification resulted in efficient CPPs; the hexaarginine derivative was the best. The cellular uptake of the peptides was examined by two different techniques, which completed each other and gave mechanistic insight about the internalisation. The internalisation of the conjugates was rapid and showed diffuse distribution already at low concentration. These findings may refer to either the direct translocation or the release from vesicles after vesicular transportation. Conjugate of this construct with antitumor drugs showed cytostatic activity on different cell lines.

## Materials and methods

All amino acid derivatives, and Rink-amide MBHA resins were purchased from IRIS Biotech GmbH (Marktredwitz, Germany), whereas *N*,*N*–diisopropylethylamine (DIEA), 1–hydroxybenzotriazole (HOBt), *N*,*N*'–diisopropylcarbodiimide (DIC), trifluoroacetic acid (TFA), 1.8–diazabicyclo[5.4.0]undec–7–ene (DBU), thioanisole, 5(6)–carboxyfluorescein (Cf), 5(6)–carboxytetramethylrhodamine (Rh) and 1,2–ethanedithiol were FLUKA (Buchs, Switzerland) and Sigma Aldrich (Budapest, Hungary) products. Dabcyl acid [4–((4-(dimethylamino)phenyl)azo)benzoic acid] was ordered from (AAT Bioquest, Inc., Sunnyvale, CA). Solvents for syntheses and purification were obtained from Molar Chemicals Kft (Budapest, Hungary). The buffers were prepared with ion exchanged distilled water.

### Synthesis of peptides

The peptides were synthesised manually by solid-phase methodology on Rink-amide MBHA resin (0.250 g, 0.69 mmol/g) using Fmoc/^*t*^Bu strategy as was described earlier (Szabó et al. 2016). After the incorporation of the last arginine into the sequence, the Fmoc protecting group was cleaved and the free N-terminal α-amino group was reacted with Dabcyl–OH using DIC-HOBt reagents (2 eqv. from each). The peptides were cleaved from the resin with 5 mL TFA-containing 0.375 g phenol, 0.25 mL distilled water, 0.25 mL thioanisole and 0.125 mL 1,2–ethanedithiol as scavengers. Crude products were precipitated by dry diethyl-ether, dissolved in 10% acetic acid and freeze-dried. The peptides were purified by RP–HPLC on a semipreparative Phenomenex Jupiter C18 column (250 × 10 mm I.D.) with 10 μm silica (300 Å pore size). Flow rate was 4 mL/min. Linear gradient elution was applied.

The coupling of fluorescence dyes (Cf or Rh) was carried out in DMF using DIC-HOBt coupling reagents in 1.1 eqv. to the peptide. The conjugate was isolated from the reaction mixture by RP–HPLC.

The purified compounds were characterised by analytical RP–HPLC and ESI–MS (Table 1 and Supplementary Information).

**Table 1:** Chemical characterisation of peptides

Sequence	*R*_t_^a^	ESI–MS^b^	
		*M*_cal_	*M*_meas_
*Ac*–RRRRK(*Cf*)–*NH*_*2*_	12.8	1169.8	1169.7
*Ac*–RRRRRRK(*Cf*)–*NH*_*2*_	11.8	1482.0	1482.2
*Dabcyl*–RRRRK(*Cf*)–*NH*_*2*_	12.9	1379.1	1379.0
*Dabcyl*–RRRRRRK(*Cf*)–*NH*_*2*_	12.4	1691.3	1691.4
*Dabcyl*–RRRRRRK(*Rh*)–*NH*_*2*_	15.3	1745.6	1746.1
*Ac*–RRRRRRK(*Rh*)–*NH*_*2*_	13.3	1536.8	1536.7
*Dmab*–RRRRRRK(*Cf)*–*NH*_*2*_	12.8	1587.8	1587.9
*Dabcyl*–RRRRRRK(*Suc*–*Dau*)–*NH*_*2*_	16.1	1943.0	1942.8
*Dabcyl*–RRRRK(*Suc*–*Dau*)–*NH*_*2*_	14.8^c^	1630.6	1630.3
*Dabcyl*–RRRRK(*MTX*)–*NH*_*2*_	12.4^c^	1457.7	1457.4
*Dabcyl*–RRRRRRK(*MTX*)–*NH*_*2*_	12.4^c^	1770.0	1770.0
*Dabcyl*–RRRRRRK(*Glu*_*5*_–*MTX*)–*NH*_*2*__1#	27.4^d^	2415.6	2415.1
*Dabcyl*–RRRRRRK(*Glu*_*5*_–*MTX*)–*NH*_*2*__2#	27.6^d^	2415.6	2415.1

^a^analytical RP–HPLC was done on Agilent Zorbax SB-C18 column (4.6 mm × 150 mm, 5 µm, 100 Å). The applied linear gradient elution was 0 min 0% B, 2 min 0% B, 22 min 90% B at 1 mL/min flow rate. The detection was carried on at *λ* = 220 nm

^b^ESI–MS

^c^analytical RP–HPLC was done on Jupiter C18 column (4.6 mm × 150 mm, 3 µm, 100 Å). The applied linear gradient elution was 0 min 0% B, 2 min 0% B, 22 min 90% B at 1 mL/min flow rate. The detection was carried on at *λ* = 220 nm

^d^analytical RP–HPLC was done on Jupiter C18column (4.6 mm × 250 mm, 3 µm, 300 Å), the applied linear gradient elution was 0 min 0% B, 5 min 0% B, 50 min 90% B at 0.8 mL/min flow rate. The detection was carried on at *λ* = 220 nm

### In vitro cell culturing

HL-60 (ATCC^®^ CCL-240™) human promyelocytic leukaemia cells (Collins et al. 1977; Gallagher et al. 1979) were grown in RPMI-1640 supplemented with 10% FCS, (L)–glutamine (2 mM) and gentamicin (160 µg/mL). Cells were maintained in plastic tissue culture dishes at 37 °C with a humidified atmosphere containing 5% CO_2_/95% air.

MCF-7 (ATCC: HTB-22) human breast adenocarcinoma cells were maintained in DMEM supplemented with 10% heat inactivated foetal calf serum (FCS), non-essential amino acids (NEAA), pyruvate (1 mM), l-Gln (2 mM) and gentamicin (160 μg/mL). MDA-MB-231 (ATCC:HTB-26) human triple negative breast adenocarcinoma cells were cultured in RPMI-1640 supplemented with 10% FCS, l-Gln (2 mM) and gentamicin (16 μg/mL). Cells were maintained in plastic tissue culture dishes at 37 °C with a humidified atmosphere containing 5% CO_2_/95% air.

Wild type Chinese Hamster–Ovary CHO-K1 cells, (CCL-61(ATCC), LGC Standards S.a.r.l.—France) were cultured in F12 growth medium (DMEM-F12) supplemented with 10% foetal calf serum (FCS), penicillin (100,000 IU/L), streptomycin (100,000 IU/L), and amphotericin B (1 mg/L) in a humidified atmosphere containing 5% CO_2_ at 37 °C.

Human cervical cancer-derived HeLa cells were cultured using α-minimum essential medium (α-MEM) supplemented with 10% heat-inactivated bovine serum (BS) [α-MEM( +)] and maintained at 37 °C in a humidified 5% CO_2_ atmosphere.

### Determination of the in vitro cellular uptake profile of compounds by flow cytometry

HL-60 cells were cultured as described above. To study the cellular uptake of fluorescent labelled compounds, 10^5^ cells per well were plated on 24-well plates. After 24 h incubation at 37 °C, cells were treated with the compounds solved in the corresponding serum-free media for 90 min. The cellular uptake was analysed at 1, 5 and 10 µM concentrations. Cells treated with serum-free media for 90 min were used as control. After incubation, treatment solutions were removed and the cells were treated with 100 μL trypsin for 2 min to remove the membrane proteins in order to eliminate non-specific binding of conjugates. The effect of trypsin was terminated by 900 μL HPMI (glucose, NaHCO_3_, NaCl, HEPES, KCl, MgCl_2_, CaCl_2_, Na_2_HPO_4_·2H_2_O) (Kapus et al. 1994) containing 10% foetal calf serum, and the cells were transferred from the plate to FACS-tubes. Cells were centrifuged at 216 *g* at 4 °C for 5 min and the supernatant was removed. After this procedure, cells were resuspended in 500 µL HPMI, and the intracellular fluorescence intensity of HL-60 cells was monitored (on channel FITC LP505; emission at *λ* = 505 nm; LP 505, BP 530/30) by flow cytometry (BD LSR II, BD Bioscience, San Jose, CA, equipped with 488 nm; Coherent Sapphire, 22 mW laser.) which is proportional to the cellular uptake. Data were analysed with FACSDiVa 5.0 software.

HeLa cells (8.0 × 10^4^/well) in α-MEM( +) were cultured in 24-well microplates overnight at 37 °C. Cells were treated with fluorescently-labelled peptides (2.5, 5 and 10 μM) in α-MEM( +) and α-MEM without 10% BS [α-MEM(–)], respectively, for 30 min at 37 °C. After being washed with phosphate-buffered saline (PBS), cells were incubated with 0.01% trypsin in PBS for 10 min at 37 °C. The collected cells were then centrifuged at 800 *g* for 5 min at 4 °C, washed twice with PBS, and subjected to flow cytometry analysis (Attune NxT Flow Cytometer, ThermoFisher). Each sample was analysed over 10,000 events.

### Confocal microscopy

HeLa cells (2.0 × 10^5^ cells) seeded in 35-mm glass-bottomed dishes (Iwaki) were cultured at a 37 °C incubator for 24 h. The cells were then incubated with peptide in α-MEM( +) and α-MEM(–) for 30 min at 37 °C or 4 °C. After washing with PBS( +) containing 0.5% (w/v) heparin, intracellular distribution of the fluorescently-labelled peptides was analysed without fixing using a confocal microscope (FV1000, Olympus). For time-lapse imaging, the cells were placed at 37 °C in a microchamber (MI-IBC-IF, Olympus) attached on the stage of the inverted microscope. The cells were then treated with 5 µM of *Dabcyl*–Arg_6_–Lys(*Rh*)–*NH*_*2*_ or *Dabcyl*–Arg_4_–Lys(*Cf*)–*NH*_*2*_ in α-MEM( +) or α-MEM(–). Time 0 represents the image immediately after the addition of conjugates.

### Analysis of the in vitro cytostatic activity of conjugates

The cells (HL-60 or MCF-7) were grown to confluency and were plated into 96-well plate with initial cell number of 5 × 10^3^ per well. After 24 h incubation at 37 °C, cells were treated with the compounds at 1.28 × 10^–3^–100 μM concentration range for 3 h in 200 μL final volume. Control cells were treated with serum free medium at 37 °C for 3 h. After incubation the cells were washed twice with serum free medium. For the analysis of the in vitro cytostatic effect, cells were cultured for an additional 72 h in serum containing medium. On day 4, MTT assay was carried out to determine the IC_50_ values of the compounds. Briefly, 45 μL of MTT solution was added to each well (2 mg/mL, dissolved in serum-free medium). Following the 4 h incubation, plates were centrifuged at 900 *g* for 5 min, and the supernatant was removed. The precipitated purple crystals were dissolved in 100 μL DMSO and the absorbance was determined at *λ* = 540 nm and *λ* = 620 nm using ELISA plate reader (iEMSReader, Labsystems, Finland). The percent of cytostasis was calculated using the following equation: Cytostatic effect (%) = [1 − (OD_treated_/OD_control_)] × 100; where OD_treated_ and OD_control_ correspond to the optical densities of the treated and the untreated control cells, respectively. In each case three independent experiments were carried out with four parallel measurements. The 50% inhibitory concentration (IC_50_) values were determined from the dose–response curves. The curves were defined using MicrocalTM Origin (version 9.2) software: cytostasis was plotted as a function of concentration, fitted to a sigmoidal curve, and based on this curve, the IC_50_ value was determined. IC_50_ values represent the concentration of a compound required for 50% inhibition in vitro and expressed as micromolar units.

#### Study the effect of FRET on the fluorescence signal

Digestion by trypsin was used to measure the total fluorescence of the labelled peptide. We used 50 pmol of *Dabcyl*–Arg_4_–Lys(*Cf*)–*NH*_*2*_ or *Dabcyl*–Arg_6_–Lys(*Cf*)–*NH*_*2*_ in 200 µL lysis buffer (50 mM Tris pH 7.4, 0.15 M NaCl, 1% NP40) in the presence or absence of trypsin. The samples were analysed with (one million) or without cells before and after incubation (1 h at 37 °C). NaCl was then added to the sample to obtain 1 M final concentration. The samples were then sonicated 15 min and centrifuged at 16 000 *g* for 10 min. Fluorescence was measured in the supernatants using a MOS 200 M fluorimeter (BioLogic SA). The fluorescence signal of peptides only was obtained by subtracting the fluorescence intensity of cell lysates (autofluorescence) from the fluorescence intensity of the sample.

### Absolute quantification of total internalised peptide by fluorometry

We used the quantification method described earlier (Illien et al. 2016). Briefly, we incubated one million CHO-K1 cells for 1 h at 37 °C (or 4 °C) with the fluorescent peptides *Dabcyl*–Arg_6_–Lys(*Cf*)–*NH*_*2*_, *Dabcyl*–Arg_4_–Lys(*Cf*)–*NH*_*2*_ or *Cf*–Arg_9_ at 1, 2.5, 5 and 10 µM in 500 µl DMEM-F12. After incubation with peptides and washing the cells with HBSS, 500 μL 0.05% trypsin/EDTA 0.05% (37 °C) or 500 µL pronase 0.05% in 100 mM Tris pH 7.4 (4 °C) was added for 5 min to hydrolyze the remaining extracellular peptide, the membrane-bound peptide and to detach cells. After addition of 100 µL enzyme inhibitors (complete mini at 4 °C (Roche) or trypsin inhibitor (soybean inhibitor 5 mg/mL) at 37 °C mixed with 100 μL bovine serum albumin (1 mg/mL), cells were transferred into a microtube, centrifuged, washed with 1 mL 50 mM Tris buffer pH 7.4, 0.1% BSA, and lysed in 200 μL 50 mM Tris pH 7.4, 1 M NaCl, 1% Nonident P40. The samples were then sonicated for 15 min (to homogenise samples) and centrifugated for 10 min at 16.000 *g*. Fluorescence intensity in the supernatants was monitored with a MOS 200 M fluorimeter (Biologic SAS) and the maximal intensity was detected around *λ* = 525 nm. The maximal intensity was retained for the calibration curve and for quantification of samples. The amount of internalised peptides was calculated by comparing the fluorescence intensity of the sample with the calibration curve. For the calibration curve, we prepared a range of peptide amounts (from 2 to 500 pmol) in the lysis buffer (50 mM Tris pH 7.4, 1 M NaCl, 1% Nonidet P40) in the presence of one million suspended cells. The samples were sonicated 15 min and centrifuged at 16.000 *g* for 10 min. Fluorescence intensity in the supernatants was monitored with a MOS 200 M fluorimeter (Biologic SAS, France) and the maximal intensity was detected around *λ* = 525 nm. The fluorescence signal of peptides only was obtained by subtracting the fluorescence intensity of cell lysates (autofluorescence) from the fluorescence intensity of the sample.

### Relative quantification of total internalised peptide by flow cytometry

We incubated one million CHO–K1 cells for 1 h at 37 °C (or 4 °C) with *Dabcyl*–Arg_6_–Lys(*Cf*)–*NH*_*2*_, *Dabcyl*–Arg_4_–Lys(*Cf*)–*NH*_*2*_ or *Cf*–Arg_9_ at 1, 2.5, 5 and 10 µM in 500 µL DMEM-F12. After incubation and washing the cells with HBSS, 500 μL 0.05% trypsin/EDTA 0.05% (37 °C) or 500 µL pronase 0.05% in 100 mM Tris-buffer (4 °C) was added for 5 min to hydrolyze the remaining extracellular peptide, the membrane-bound peptide and to detach cells. After addition of 100 µL enzyme inhibitors (complete mini at 4 °C (Roche) or trypsin inhibitor (soybean inhibitor 5 mg/mL) at 37 °C mixed with 100 μL bovine serum albumin (1 mg/mL). Cells were transferred into a microtube, centrifuged, washed with 1 mL 50 mM Tris-buffer pH 7.4, 0.1% BSA, and the cell pellet was suspended in 400 μL of PBS. The fluorescence of individual cells was analysed with a FACS Calibur flow cytometer. 20.000 cells were measured for each experimental condition. The mean fluorescence of a sample was obtained by subtracting the autofluorescence of cells from the measured mean fluorescence of cells in the presence of the peptide.

## Results and discussion

### Synthesis of peptides

The Dabcyl group is frequently used in FRET-based enzyme substrate studies as dark quencher (Tompa et al. 2004; Bánóczi et al. 2008a). When the internalisation of our substrate peptide with or without Dabcyl group was measured it turned out that the presence of the Dabcyl group increased dramatically the internalisation. Similar observation was demonstrated by Roloff et al. (2018) who prepared Dabcyl-containing nanoparticles. This kind of chemically modified nanoparticle with high density of Dabcyl group has very impressive, it has significantly increased cellular uptake profile compared to the undecorated one. It is also worth to note, this enhanced cellular uptake stems from the high density of Dabcyl group, the chemical modification alone cannot be influenced this effect. Based on these results a set of oligoarginine peptides were designed to establish the positive effect of Dabcyl group on the cellular uptake. The peptides were synthesised using Fmoc/^*t*^Bu strategy. In all cases the modification of α-amino group was performed on the resin and the ε-amino group of the C-terminal lysine was reacted with fluorescence dye in solution using DIC/HOBt coupling reagents.

Dabcyl group was attached to the N-terminal Arg, thus a lysine was built in at the *C*-terminus of the oligoarginine peptides. Its ε-amino group was used to conjugate the fluorescent dye, Cf or Rh. Conjugates with antitumor drugs were synthesised on the resin (*Dabcyl*–RRRRRRK(*Glu*_*5*_–*MTX*)–*NH*_*2*_) using Mtt-protected lysine or in solution (*Dabcyl*–RRRRRRK(*Suc*–*Dau*)–*NH*_*2*_).

### Cellular uptake of peptides containing Dabcyl group

**Fig. 1:**
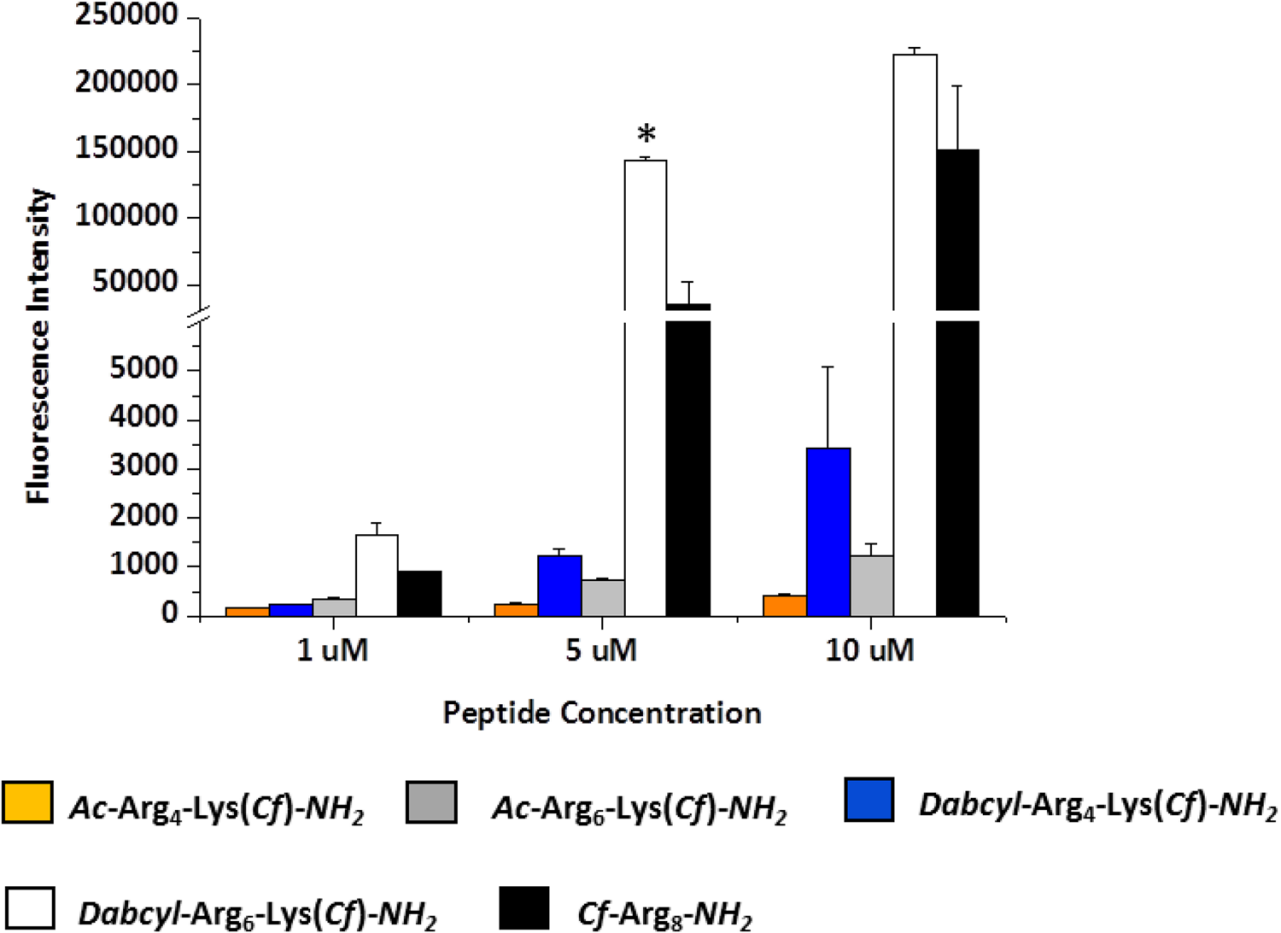
Effect of the presence of Dabcyl group on the internalisation of peptides into HL-60 cells. *Dabcyl*–Arg_4_–Lys(*Cf*) has higher cellular uptake than tetra- and hexaarginine. *Dabcyl*–Arg_6_–Lys(*Cf*) showed higher internalisation than octaarginine. The cells were treated with the peptides at 1, 5 and 10 μM concentration at 37 °C for 90 min. Then they were trypsinised and the fluorescence of cells was studied by flow cytometry. Differences between the *Dabcyl*–Arg_6_–Lys(*Cf*) and *Cf*–Arg_8_ were determined by Student’s *t* test (*p < 0.05). Data represents the mean ± standard deviation (SD) (*n* = 3)

The internalisation ability of the peptides was first determined by flow cytometry on different cells lines. HL-60 cells were treated with the peptides at 1, 5 and 10 μM concentration at 37 °C for 90 min and the fluorescence of cells was measured. In well accord with the literature the hexaarginine showed higher internalisation, than the tetraarginine (Fig. 1). It is well known that the increasing number of arginine residues enhances the internalisation up to 12 residues (Mitchell et al. 2000; Futaki et al. 2001). The Dabcyl group that was coupled to the N-terminus of tetraarginine increased the cellular uptake (Fig. 1). This derivative showed higher cellular uptake than the hexaarginine (Fig. 1). Although the fluorescence intensity of the cells was 1.6 and 2.8 times higher, later it turned out that the Dabcyl group quenches significantly the fluorescence of Cf in this construct (Fig. 11). However, this derivative is more effective than hexaarginine, the ratio of the cellular uptake of these conjugates cannot be compared.

The Dabcyl group had more remarkable effect on the internalisation of hexaarginine (Fig. 1). This construct can be taken up more effectively by HL-60 cells than octaarginine a well-known cell-penetrating peptide already at 5 μM concentration. Its cellular uptake was 4 and 1.5 times higher than that of octaarginine (at 5 and 10 μM, respectively). Dabcyl group has two benzene rings with extended delocalisation via azo-bond and a dimethylamino group, a hydrophilic group. Although there are several examples for N-terminal modification of oligoarginines to improve their penetration, but these commonly mean the coupling of hydrophobic entity (e.g. fatty acids (Pham et al. 2004; Kim et al. 2006; Lee and Tung 2010; Katayama et al. 2011; Swiecicki et al. 2015) or short peptide sequence with hydrophobic amino acids (Takayama et al. 2009, 2012; Okuda et al. 2019). Furthermore, there are several examples for insertion of Trp and thus aromatic ring into the oligoarginine sequence may increase the penetration (Mandal et al. 2011; Rydberg et al. 2012; Jobin et al. 2015). However, the effect was position-dependent: the uptake was higher in case of central position as compared with the N-terminus.To the best of our knowledge, modification with small aromatic group of the CPPs in order to increase their internalisation is not a best-known method.

**Fig. 2:**
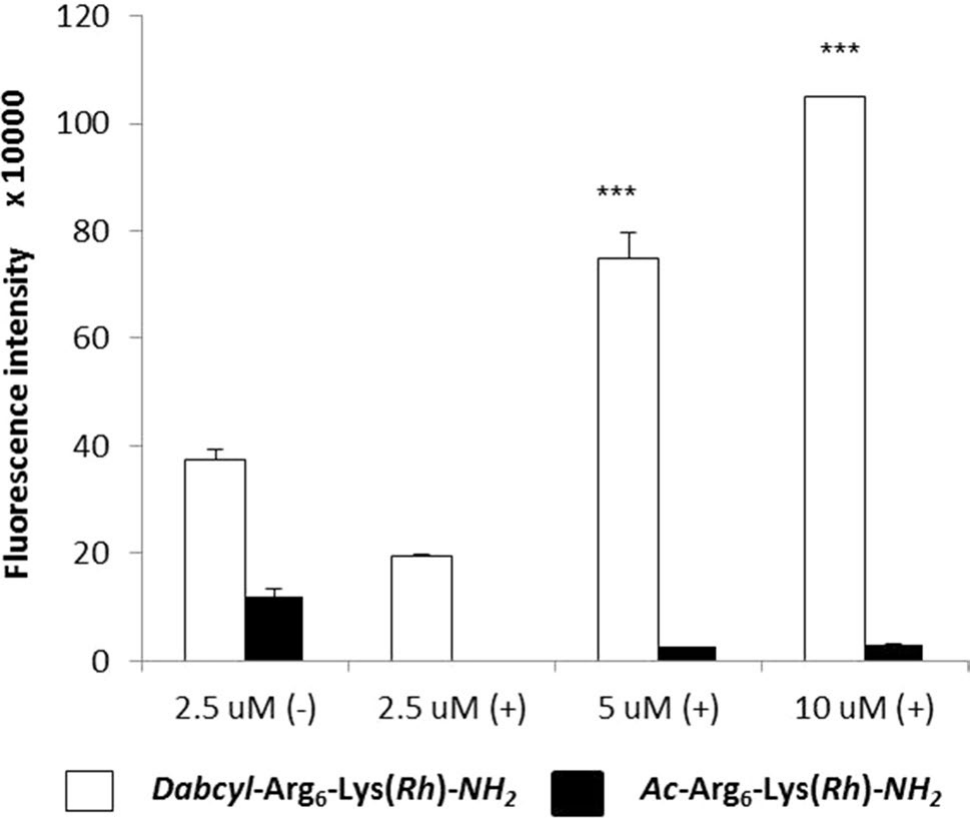
Cellular uptake of *Dabcyl*–Arg_6_–Lys(*Rh*)–*NH*_*2*_ and *H*–Arg_6_–Lys(*Rh*)–*NH*_*2*_ by HeLa cells. The cells were treated with the solution of peptides at 2.5, 5 and 10 μM concentration for 30 min in medium with serum ( +) or without serum (−). Differences between the *Dabcyl*–Arg_6_–Lys(*Rh*) and *Ac*–Arg_6_–Lys(*Rh*) were determined by Student’s *t* test (***p < 0.001) Data represent the mean ± standard deviation (SD) (*n* = 3)

Hexaarginine peptides containing Rh as a fluorescent dye was studied on HeLa cells by flow cytometry too. In these experiments the influence of serum on the cellular uptake was examined. HeLa cells were treated with the solution of conjugate (at 2.5, 5 and 10 μM concentration) in medium or without serum for 30 min (Fig. 2).

On HeLa cells, the cellular uptake profile of the Rh containing peptides (with or without Dabcyl group) was similar to Cf labelled peptide on HL-60 cells, suggesting that the fluorescence dye has no marked influence on the internalisation. In the presence of serum, *Dabcyl*–Arg_6_(*Rh*)–*NH*_*2*_ appeared to be markedly pronounced, while its acetylated derivative was undetectable in the studied lowest concentration (Fig. 2). In the absence of serum, *Dabcyl*–Arg_6_(*Rh*)–*NH*_*2*_ showed twice as much higher internalisation compare to serum ( +) one (2.5 µM). Its cellular uptake was 3 times higher than hexaarginine’s under this condition. Thus the presence of serum dramatically decreased the cellular uptake of peptides, which is in good correlation with the literature (Kosuge et al. 2008). Peptides can bind to the proteins of serum and thus their effective concentration decreases.

### Mechanism of internalisation

**Fig. 3:**
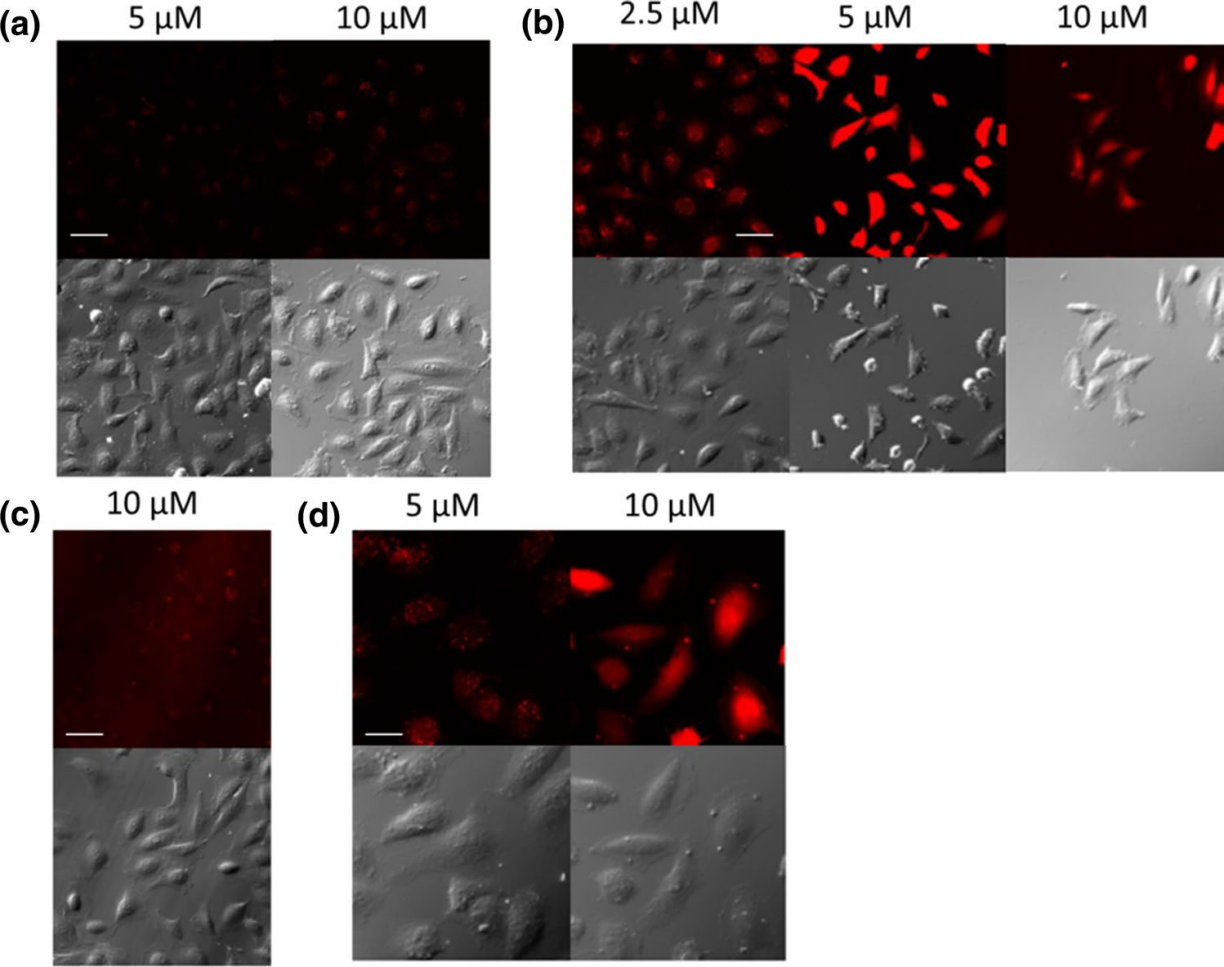
Internalisation of peptides into HeLa cells. The HeLa cells were incubated with **a** *Ac*–Arg_6_–Lys(*Rh*)–*NH*_*2*_, **b** *Dabcyl*–Arg_6_–Lys(*Rh*)–*NH*_*2*_ at different concentrations in α-MEM(–) medium and **c** *Ac*–Arg_6_–Lys(*Rh*)–*NH*_*2*_, **d** *Dabcyl*–Arg_6_–Lys(*Rh*)–*NH*_*2*_ at different concentrations in α-MEM( +) medium for 30 min at 37 °C and the fluorescence of Rh was detected. [× 20 (**a**, **b**, **c** scale bar 50 μm) and × 40 (**d**, scale bar 100 μm) enlarge]

The mechanism of cellular uptake was monitored by confocal laser scanning microscopy. HeLa cells were treated with the peptides in serum free medium for 30 min (Fig. 3).

In case of *Ac*–Arg_6_–Lys(*Rh*)–*NH*_*2*_, weak fluorescence signal was detected even at high concentration (10 μM) (Fig. 3a) in comparison with its Dabcyl containing derivative (Fig. 3b). The attachment of Dabcyl group to the N-terminus of hexaarginine dramatically increased its cellular uptake (Fig. 3b). This derivative internalised very intensively into the cells even at 2.5 μM concentration. At 10 μM concentration, *Dabcyl*–Arg_6_–Lys(*Rh*)–*NH*_*2*_ treated cells had higher fluorescence intensity than *Ac*–Arg_6_–Lys(*Rh*)–*NH*_*2*_-treated ones. It is worth to note, not only the extent of the internalisation of the peptides (with or without Dabcyl group) was different, but also their intracellular distribution (Fig. 3a, b). *Ac*–Arg_6_–Lys(*Rh*)–*NH*_*2*_ showed punctate distribution at 5 and 10 μM, while the Dabcyl-modified hexaarginine showed diffuse distribution pattern already at 2.5 μM. The morphology of the cells was dramatically changed due to the treatment of *Dabcyl*–Arg_6_–Lys(*Rh*)–*NH*_*2*_ at higher concentrations (5 and 10 μM, Fig. 3b). In addition, significant decrease in the cell number was observed, which is presumably related to the cell death caused by the conjugates and thus their detachment from the surface. This phenomenon is observed at both concentrations, but is really expressed at higher, 10 μM concentration. Interestingly, the presence of serum (α-MEM ( +)), is altered the rate and the manner of cellular uptake (Fig. 3c, d). Similar to *Ac*–Arg_6_–Lys(*Rh*)–*NH*_*2*_, which produced a weak signal at 10 μM (Fig. 3c), the cellular uptake of *Dabcyl*–Arg_6_–Lys(*Rh*)–*NH*_*2*_ also decreased. However, it was well detectable at 5 μM (Fig. 3d), this punctate fluorescence signal was significantly lower than that measured in the absence of serum at 5 μM concentration. At the highest (10 µM) concentration diffuse cytosolic localisation of *Dabcyl*–Arg_6_–Lys(*Rh*)–*NH*_*2*_ was observed. Under this condition no morphological changes was observed.

This observation suggested, there is a threshold in the concentration above which the localisation and distribution of peptides changed. The newly appearing intense diffuse fluorescence signal may be derived from the amount of directly penetrated peptide and/or from the vesicles released peptides. In case of *Ac*–Arg_6_–Lys(*Rh*)–*NH*_*2*_, this threshold concentration is between 5 and 10 μM. Attachment of Dabcyl group decreased this concentration under 5 μM, but significantly diffuse signal can be also observed even at *c* = 2.5 μM (Fig. 3c). The presence of serum decreased the concentration of free peptide as well as the level of the cellular uptake, thus increased this threshold concentration. In the absence of serum, the *Dabcyl*–Arg_6_–Lys(*Rh*)–*NH*_*2*_ resulted in morphological changes and significantly decreased the cell number, suggesting it has also effect on the cell-viability. In the presence of serum this effect was eliminated, suggesting its concentration dependency.

**Fig. 4:**
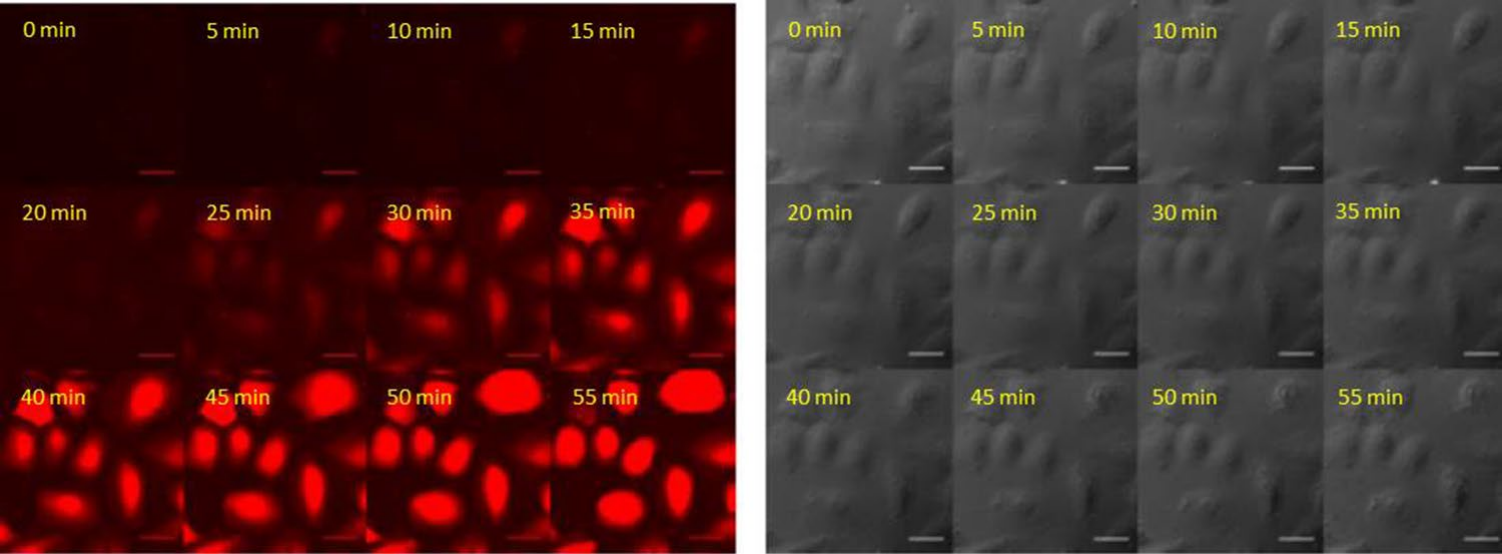
Time dependence of the internalisation of peptide into HeLa cells in the presence of serum. The HeLa cells were incubated with *Dabcyl*–Arg_6_–Lys(*Rh*)–*NH*_*2*_ at 5 μM concentrations in α-MEM ( +) at 37 °C (× 40 enlarge, scale bar 100 μm) (Time-laps imaging of every 5 min, from 0 to 55 min). The fluorescence of Rh was detected

The time-dependence of the internalisation was studied at 37 °C. The cells were maintained on the stage of the microscope and pictures were recorded from the same spot in every 5 min. The cells were treated in α-MEM ( +) at 5 μM (Fig. 4).

After 5 min only weak fluorescence signal was detected and 25 min was needed to get well detectable signal with diffuse cytosolic distribution. This result seems to be inconsistent with the result presented in Fig. 3d, where punctate fluorescence signal was demonstrated. This discrepancy can be explained as an artificial redistribution of the peptide caused by vesicular disruption due to repeated laser irradiation (Matsushita et al. 2004; Maiolo et al. 2004).

**Fig. 5:**
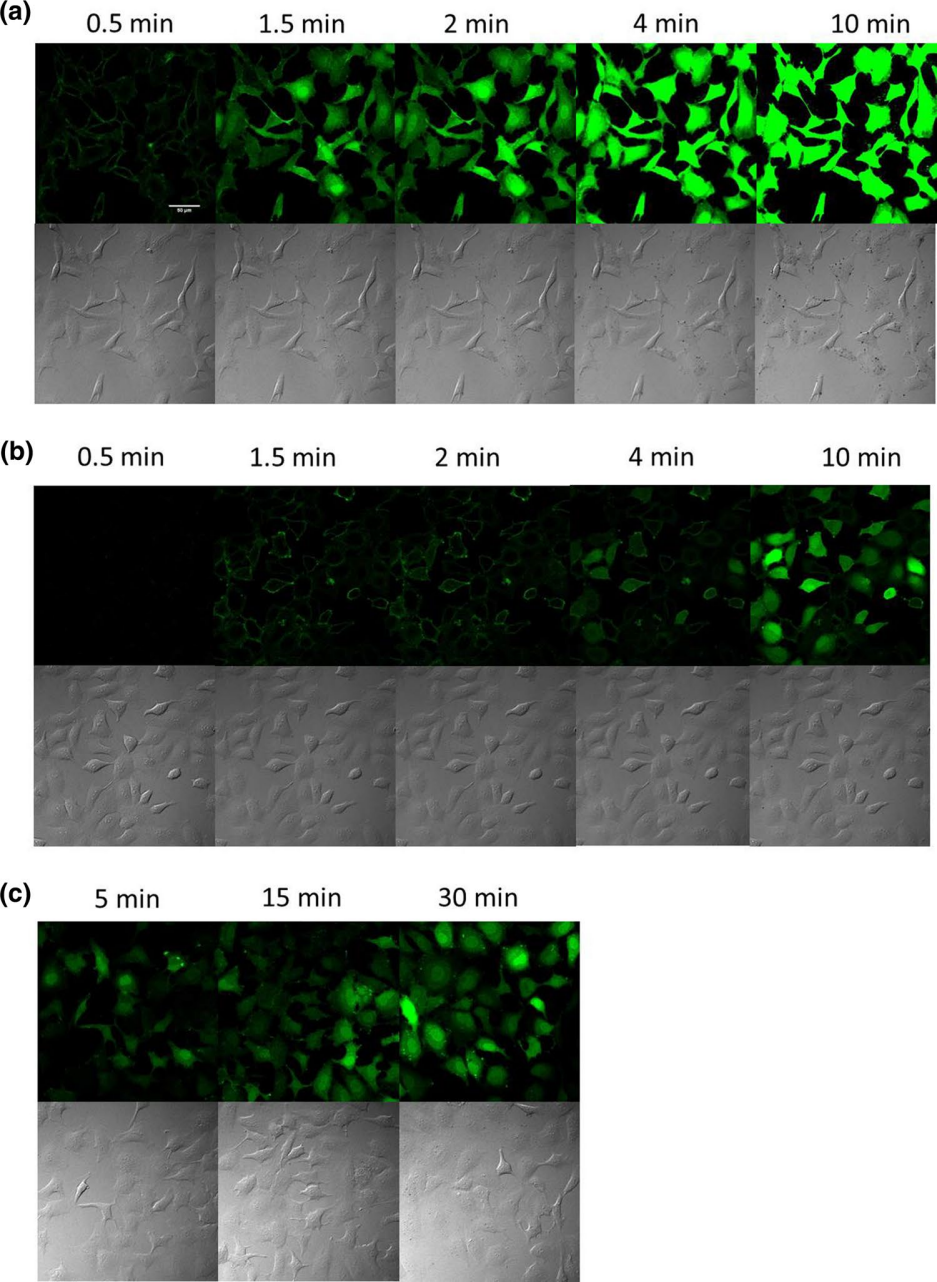
The role of macropinocytosis and other endocytosis in the internalisation of *Dabcyl*–Arg_6_–Lys(*Cf*)–*NH*_*2*_ into HeLa cells. **a** The HeLa cells were incubated with *Dabcyl*–Arg_6_–Lys(*Cf*)–*NH*_*2*_ at 5 μM concentrations in α-MEM(–) at 37 °C (× 20 enlarge) or **b** HeLa cells were preincubated with EIPA (*c* = 100 μM) for 30 min then was treated with *Dabcyl*–Arg_6_–Lys(*Cf*)–*NH*_*2*_ at 5 μM concentrations in α-MEM(–) at 37 °C (× 20 enlarge); **c** the HeLa cells were incubated with *Dabcyl*-Arg_6_-Lys(*Cf*)-*NH*_*2*_ at 5 μM concentrations in α-MEM(–) at 4 °C (× 20 enlarge). Scale bar: 50 µm. The fluorescence of Cf was detected

As the serum has high effect on the internalisation of peptides, in the further experiments *Dabcyl*–Arg_6_–Lys(*Cf*)–*NH*_*2*_ was used at 5 μM concentration in serum free (–) medium (Fig. 5a) and the internalisation of this peptide was studied in shorter period of time. It seems that the binding of the *Dabcyl*–Arg_6_–Lys(*Cf*)–*NH*_*2*_ to the cell membrane was very fast (0.5 min) and its internalisation is occurred within 1.5 min. The morphology of cells dramatically changed by treatment of *Dabcyl*–Arg_6_–Lys(*Cf*)–*NH*_*2*_, which might refer to its cytotoxicity. This observation is in good correlation with in the Fig. 3b demonstrated results. In case of arginine-rich CPPs the micropinocytosis is suggested as an important internalisation pathways (Nakase et al. 2004, 2007). For studying the role of this pathway in the internalisation of our peptides, cells were preincubated with 5–(*N*–ethyl–*N*–isopropyl)amirolide (EIPA) the well-known macropinocytosis inhibitor (Meier et al. 2002; Koivusalo et al. 2010) for 30 min. In this case very different internalisation pattern was detected (Fig. 5b). The penetration was slow and its level decreased. After 2 min only few cells had intracellular fluorescence signal and the peptide mainly exhibited membrane bound localisation. The cell morphology was not changed. If the cells were treated at 4 °C, the intracellular localization would be altered again (Fig. 5c). After 5 min, diffuse distribution was detected and all of the cells had significant fluorescence signal, but lower than at 37 °C. Increasing the incubation time (till 30 min) the amount of cellular uptake was increased.

Based on these results, it seems that the internalisation of *Dabcyl*–Arg_6_–Lys(*Cf*)–*NH*_*2*_ is very fast and may happen via two pathways; direct penetration and endocytosis at 37 °C. The treatment with EIPA decreased markedly the amount of internalised peptide at 37 °C, suggesting involvement of micropinocytosis in the internalisation of the peptide. It should be mentioned that the reported effects of EIPA were rather different; the cellular uptake of nonaarginine was dramatically increased by EIPA (peptide was used at 5 μM or higher concentration) (Duchardt et al. 2007), while the internalisation of octaarginine (c(peptide) = 10 μM) was inhibited on HeLa cells (Nakase et al. 2004).

Thus the internalisation pathway of *Dabcyl*–Arg_6_–Lys(*Cf*)–*NH*_*2*_ may be different. Cellular uptake study at 4 °C demonstrated that the amount of internalised peptide is similar to that of entered the cell in the presence of EIPA (Fig. 5b, c). In both cases the cellular uptake was lower and the peptide had essentially no effect on the cell morphology that was seen after short period of time (1.5 min) at 37 °C. It seems this effect depends on the intracellular concentration of peptide, which was reduced by the low temperature or by the inhibitor. As very fast appearance of diffuse signal was seen at 37 °C already after 1.5 min (Fig. 5a), which was missing in case of macropinocytosis inhibition, it may refer that peptide can be released form the macropinosomes and diffuses in the cytosol.

**Fig. 6:**
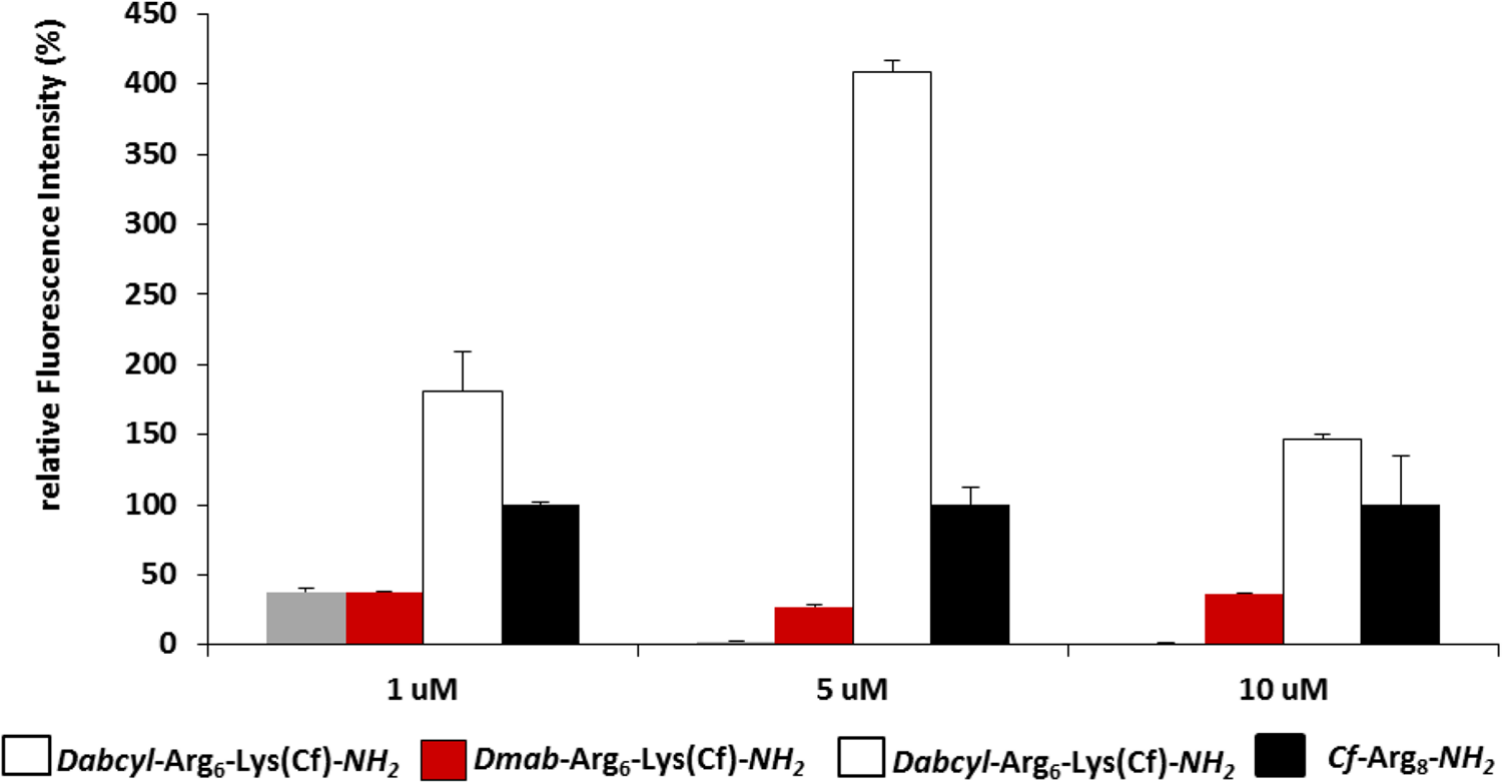
Effect of Dmab group on the internalisation of hexaarginine into HL-60 cells. The cells were treated with the peptides [*Dmab*–Arg_6_–Lys(*Cf*)–*NH*_*2*_ (white column) and *Cf*–Arg_8_–*NH*_*2*_, (black column)] at 1, 5 and 10 μM concentration at 37 °C for 90 min. Then they were trypsinized and the fluorescence of cells was studied by flow cytometry. The fluorescence intensities were normalised to fluorescence intensity of cells that were treated with *Cf*–Arg_8_ (this fluorescence intensity is 100%)

Since in the Dabcyl group, two benzene rings are linked by azo-bond, we wanted to clarify whether one aromatic ring might be sufficient to enhance the cellular uptake or not. Therefore, conjugate with *p*–dimethylaminobenzoic acid (Dmab) (*Dmab*–Arg_6_–Lys(*Cf*)–*NH*_*2*_) was also synthesised. The internalisation of this construct was studied by flow cytometry on HL-60 cells (Fig. 6). It seemed that this group could also increase the cellular uptake, but its effect is less pronounced as compared to the Dabcyl moiety. This may refer to the necessity of the two benzene rings of Dabcyl group for the efficient internalisation.

**Fig. 7:**
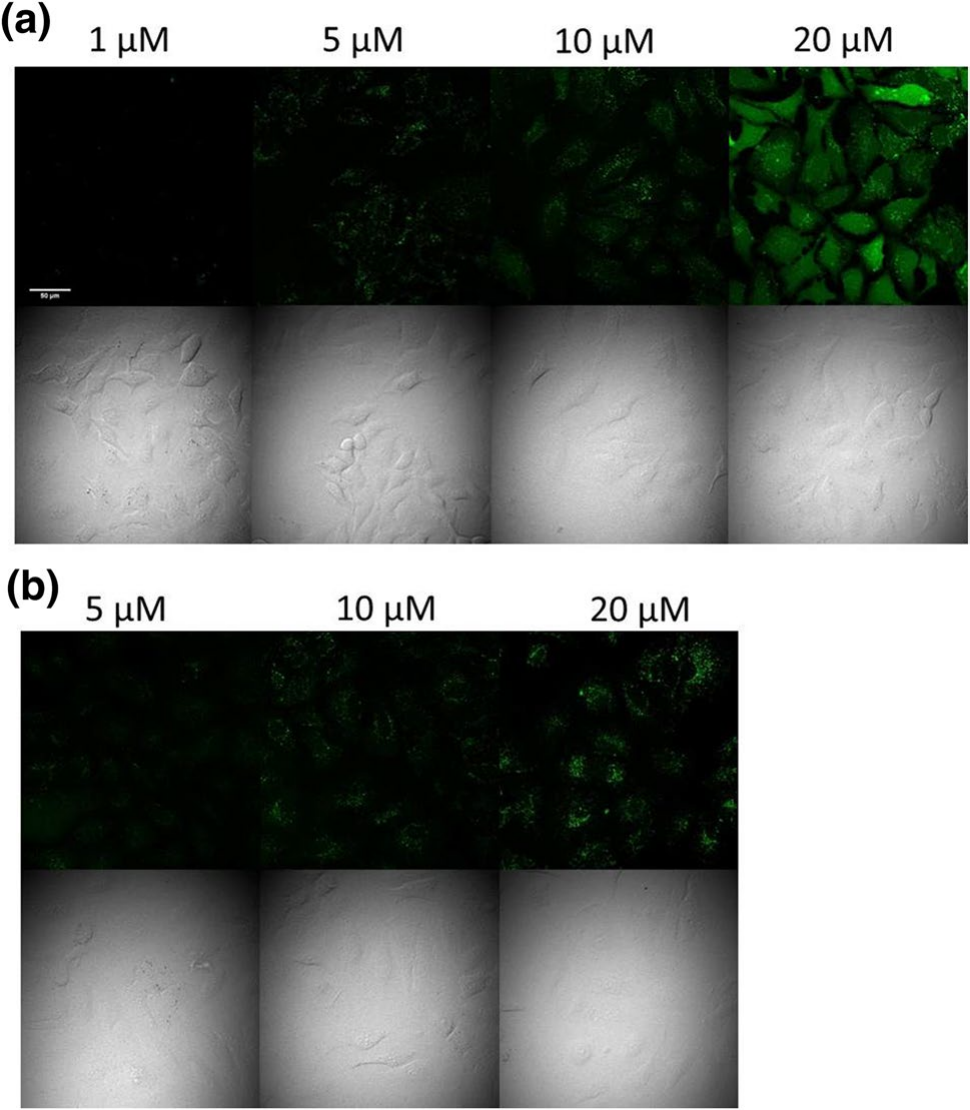
Internalisation of peptide with Dmab group into HeLa cells. The HeLa cells were incubated with *Dmab*–Arg_6_–Lys(*Cf*)–*NH*_*2*_ at different concentrations in **a** α-MEM(–) or **b** α-MEM( +) medium at 37 °C (× 20 enlarge). The fluorescence of Cf was detected

The cellular localisation of this peptide was examined by confocal fluorescence microscopy (Fig. 7). As flow cytometry, this experiment also showed that this construct cannot penetrate so efficiently than the Dabcyl-containing one. At *c* = 1 and 5 μM the cells had very low fluorescence intensity and only punctate distribution was detected. Diffuse signal could be observed only at 10 μM concentration (Fig. 7a). The vesicles were detectable even at higher concentration (20 μM). In the present of serum, the internalisation was dramatically reduced and only punctuate signal was presence even at *c* = 20 μM (Fig. 7b). This compound had no effect on the cell viability, the morphology was not changed. It should be mentioned that the number of cells was the same at all concentrations. Our data suggest that incorporation of an aromatic ring with positively charged dimethyl amino group at the *N*-terminus can increase the internalisation of an oligoarginine, although its effect is not so pronounced as compared with that of Dabcyl group.

The fluorescence microscopy images show that it may use the similar machinery to enter into the cells, than the Dabcyl-modified hexaarginine, and it seems that the threshold concentration of diffuse distribution in case of *Dmab*–Arg_6_–Lys(*Cf*)–*NH*_*2*_ more than 10 μM.

**Fig. 8:**
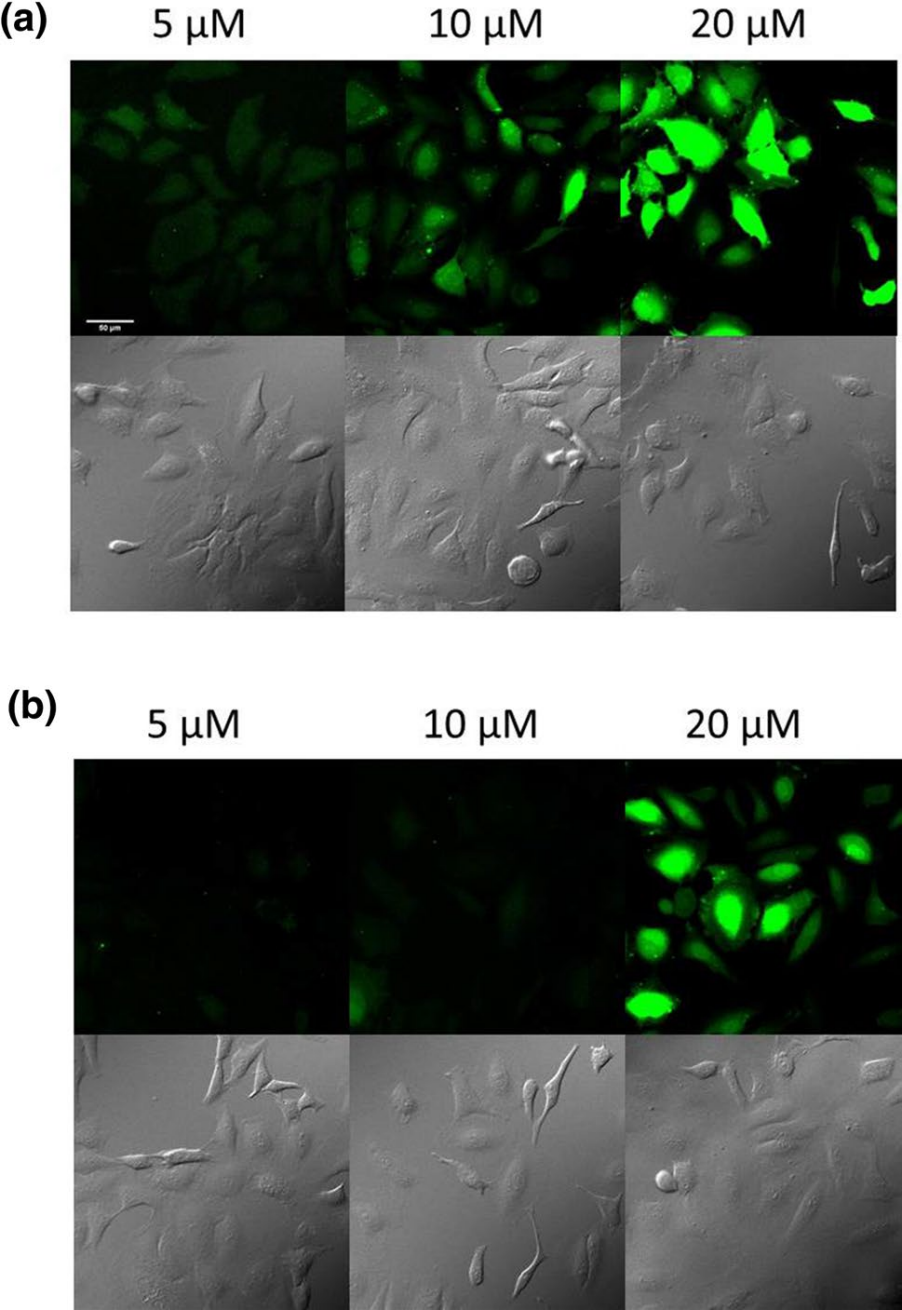
Internalisation of *Dmab*–Arg_6_–Lys(*Cf*)–*NH*_*2*_ into HeLa cells at 4 °C. The HeLa cells were incubated with *Dmab*–Arg_6_–Lys(*Cf*)–*NH*_*2*_ at different concentrations in **a** α-MEM(–) or **b** α-MEM( +) medium at 4 °C (× 20 enlarge)

Its internalisation was also studied at 4 °C (Fig. 8). The conjugate had diffuse distribution pattern with nuclear localisation at higher concentration (10 and 20 μM). In the presence of serum (Fig. 8b) the rate of cellular uptake decreased, but the distribution profile was very similar.

These findings suggest that *Dmab*–Arg_6_–Lys(*Cf*)–*NH*_*2*_ conjugate can also penetrate directly, but this way was totally suppressed at 37 °C. These results are in harmony with observation that the mechanism of internalisation in case of arginine-rich peptides depends on many factors (Fretz et al. 2007). It underlines the observation on the inhibition of vesicular transport by the low temperature. Thus, the direct penetration becomes the pathway of the cellular uptake.

### The effect of FRET on the fluorescence signal

**Fig. 9:**
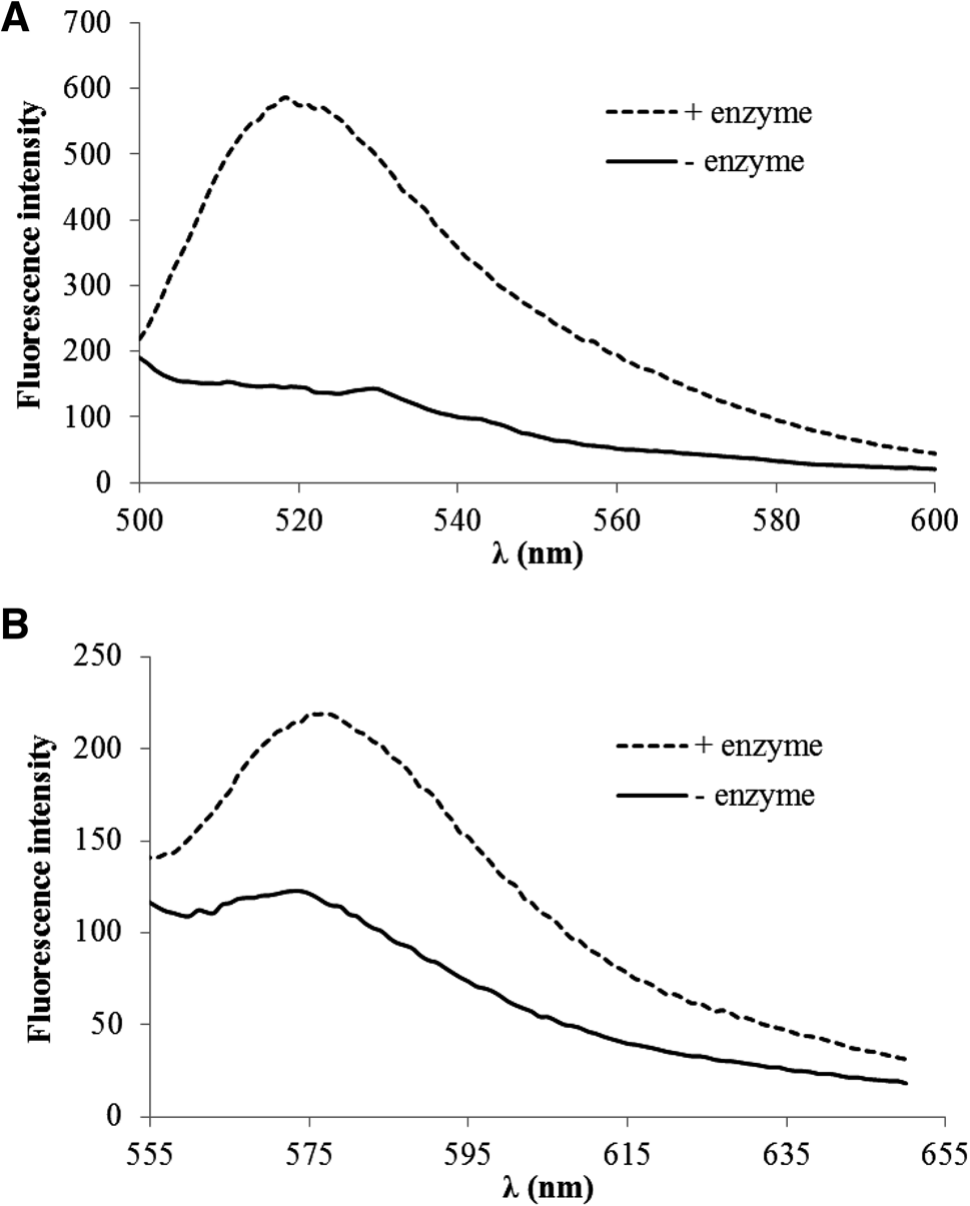
The effect of digestion of peptides by trypsin on fluorescence intensity. Hexaarginine with **A** Dabcyl–Cf and **B** Dabcyl–Rh pair were digested by trypsin at 10 μM concentration. The fluorescence of peptides was recorded in buffer (− enzyme) and in the presence of trypsin (+ enzyme) after 10 min

The Dabcyl group is well-known as a black quencher for Cf fluorescence in FRET system (Tyagi et al. 1998; Tsuji et al. 2013; Moss et al. 2016). This phenomenon may affect significantly the interpretation of results based on the fluorescence intensity. For studying the FRET in our constructs *Dabcyl*–Arg_6_–Lys(*Cf*)–*NH*_*2*_ (*c* = 10 μM) were digested by trypsin (Fig. 9A) for 10 min. After the digestion the fluorescence intensity was increased dramatically (4 times), which means that in the intact peptide there is highly efficient FRET. In order to decrease the FRET, another fluorescence dye, 5(6)–carboxytetramethylrhodamine, was used instead of Cf. This fluorophore can be excited at longer wavelength (*λ* = 535 nm). The cleavage of the peptide by trypsin increased the fluorescence intensity too, but this change was smaller (1.8 times) (Fig. 9B). This could mean that the overlapping of the absorbance of Dabcyl group is not so efficient with the emission of Rh moiety.

### Determination of the amount of internalised peptides by fluorometry

**Fig. 10:**
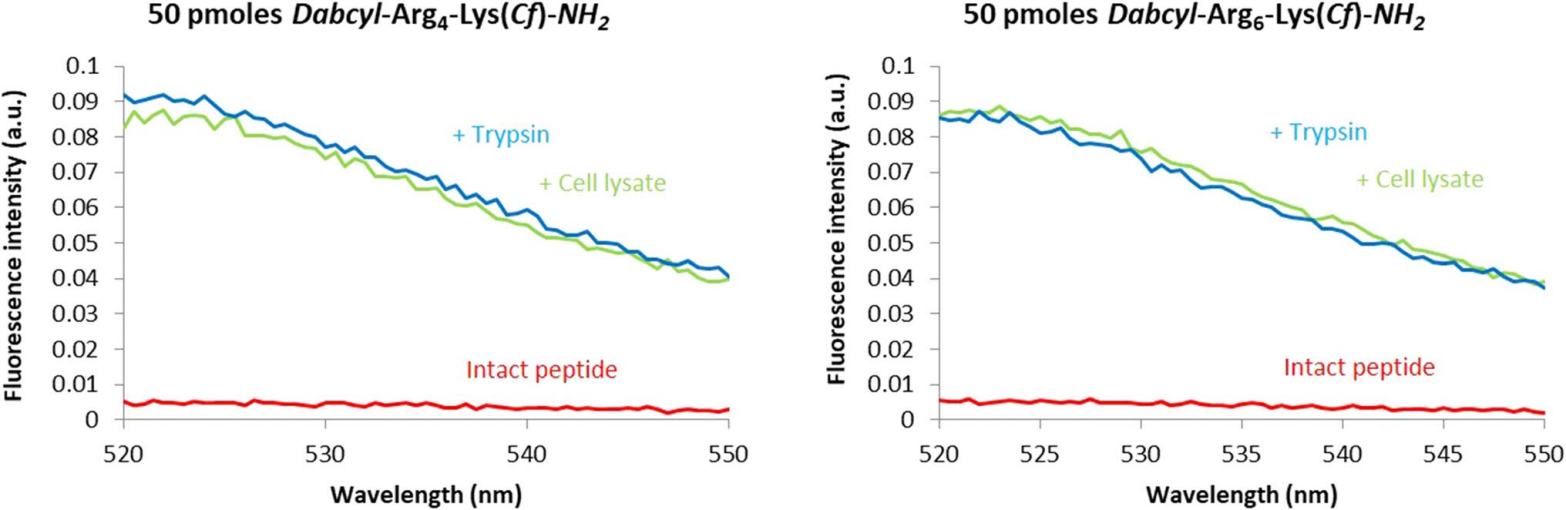
Digestion of peptides by trypsin or by cell lysates. Fluorescence was recorded in supernatants after treatment of intact peptide with trypsin and/or cell lysates and centrifugation. The fluorescence signal of peptide only was obtained by substracting the fluorescence intensity of cell lysates (autofluorescence) from the fluorescence intensity of the sample

**Fig. 11:**
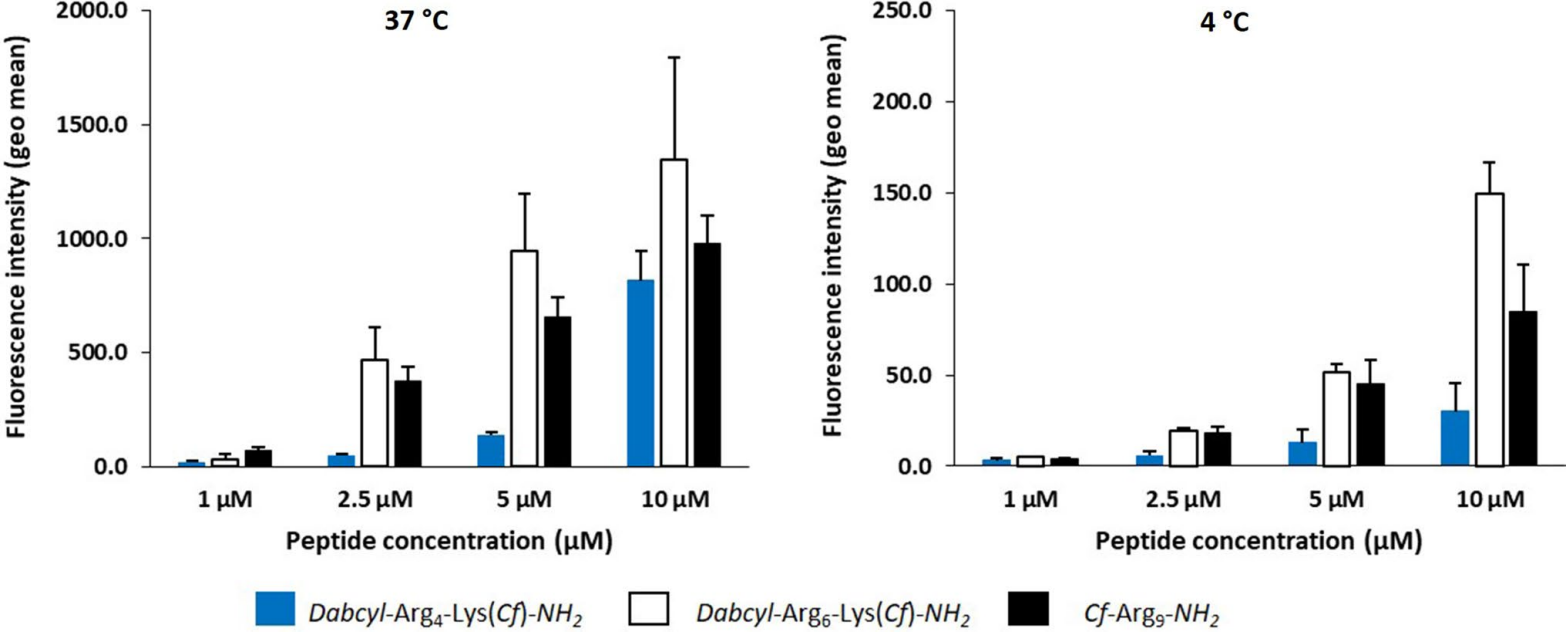
Internalisation efficacy at 37 °C and 4 °C of tetraarginine, hexaarginine and nonaarginine analysed by flow cytometry. Cells were incubated at 37 °C and 4 °C with different concentrations of peptides. After incubation, cells were treated with trypsin to detach cells and remove membrane-bound peptides, before washings and cell fluorescence analysis by flow cytometry

The FRET between the Dabcyl group and fluorescence dye means that the fluorescence signal highly depends on the possible cleavage of peptides in cells. This effect makes very difficult the interpretation of results of flow cytometry and confocal microscopy experiments. In a recent paper (Illien et al. 2016) quantification of the amount of internalised peptides by fluorometry was described. In this method, after treatment with the peptides, cells are lysed and the fluorescence intensity of the cell lysate is measured under conditions allowing recovery of the total fluorescence signal, which is then compared to a calibration curve to determine the absolute quantity of intracellular peptide (Illien et al. 2016). Thus, the internalisation ability of different peptides can be compared. We first checked that full cleavage of the peptides can be obtained by enzymes released during cell lysis. First, cells were treated with *Dabcyl*–Arg_4_–Lys(*Cf*)–*NH*_*2*_ or *Dabcyl*–Arg_6_–Lys(*Cf*)–*NH*_*2*_, then lysed in the presence or absence of trypsin. In the cells, enzymes released during cell lysis cleaved the peptide and resulted in the maximal fluorescence signal (Fig. 11), which was similar with the addition of exogenous trypsin. Thus, the release of enzymatic activities during cell lysis are sufficient to cleave the peptides resulting in the recovery of the whole fluorescence corresponding to the total intracellular concentration of the peptides (Fig. 10). The fluorescence intensity of lysates was then systematically measured and the amount of peptide was determined using the calibration curve done in parallel, as described (Illien et al. 2016).

CHO-K1 cells were treated with peptides either at 37 °C and 4 °C to study the mechanism of internalisation and to determine the intracellular peptide concentration (Illien et al. 2016).

The internalisation was analysed both by flow cytometry and by fluorometry in parallel with the evaluation of endocytosis and translocation contributions in the internalisation process. In intact cells (flow cytometry) both native and cleaved peptides can coexist at 37 °C, and only native peptide at 4 °C. When lyzing cells after internalisation (fluorometry), we had access only to cleaved peptide, thus to the total internalised peptide at 37 °C or 4 °C, whatever the internalisation pathway.

In flow cytometry experiments, at 37 °C the internalisation of hexaarginine was higher at all tested concentrations compared to nonaarginine and tetraarginine (Fig. 11). According to the peptide concentration, the internalisation of hexaarginine was 1.7, 10, 7 and 1.7 times higher (at 1, 2.5, 5 and 10 µM, respectively) than that of tetraarginine. When cells were treated at 4 °C a different picture was seen (Fig. 11). According to the extracellular peptide concentration, the internalisation of hexaarginine was only 1.6, 4, 4 and 5 times higher (at 1, 2.5, 5 and 10 µM, respectively) than that of tetraarginine.

The cellular uptake of nonaarginine, a well-known cell-penetrating peptide, was also studied. At 37 °C it showed lower internalisation than hexaarginine but higher than tetraarginine. Interestingly, at the highest concentration (10 μM) the tetraarginine was similarly internalised as nonaarginine. At 4 °C, hexaargine and nonaarginine were internalised to the similar extent up to 10 μM, while tetraarginine internalisation was always lower. In this latter case, cells maintained at 4 °C from the incubation with peptides to flow cytometry analysis. Thus, intracellular enzymes are inactive and the fluorescence measured only results from the intact peptide residual fluorescence, which is about 10–12 times lower in intensity than for the cleaved peptide (not shown).

In the flow cytometry conditions when cells have been incubated with the peptides at 37 °C, the peptide can be present in the cleaved form (endocytosis) or the native form (translocation and/or endosomal escape). Thus, the fluorescence intensity for the Dabcyl-containing peptides can reflect a very high concentration of native peptide, a lower concentration of cleaved peptide or a combination of both in intact cells. In the absence of the intramolecular Dabcyl quencher, the fluorescence signal could also be underestimated because of acidic pH environment (endosomes) or high local peptide concentrations. It is thus very difficult to compare the internalisation efficiency of peptides in these conditions.

Therefore, we used the method previously reported to quantify in an absolute manner, fluorescent peptides in cells (Illien et al. 2016). For the design of these experiments, after incubation with peptides, cells were treated with trypsin (37 °C) or pronase (4 °C) to remove membrane-bound peptides. After washings, cells were lyzed to allow enzymatic degradation (dequenching) of internalised peptides and recovery of full fluorescence that corresponds to the total quantity of internalised peptide. At 37 °C, the peptide quantity reflects internalisation potentially by both endocytosis and translocation, while at 4 °C, only translocation can occur. Results are shown in Table 2. Regarding hexaarginine and nonaarginine, the data (translocation/endocytosis ratio) indicate that these peptides preferentially internalise by endocytosis already at low μM concentrations. The situation is different for tetraarginine that internalises principally by direct translocation up to *c* = 5 μM.

**Table 2:** Quantification of endocytosis and translocation of tetraarginine, hexaarginine derivatives and nonaarginine on CHO-K1 cells by fluorometry (Illien et al. 2016)

	[peptide] (μM)	Total 37 °C^a^ (pmoles) Endocyt. + Transloc.	Total 4 °C^a^ (pmoles) Transloc.	Unpaired *T*-test (37 °C vs 4 °C)	Total (37 °C–4 °C) (pmoles) Endocyt.	4 °C/37 °C (Fluoro)	4 °C/37 °C (Flow cyto.)
*Dabcyl*–Arg_4_–Lys(*Cf*)–*NH*_*2*_	1	0.7 ± 0.1	0.4 ± 0.06	** (*p* = 0.001)	0.3	0.57	0.17
	2.5	2.2 ± 0.2	0.9 ± 0.1	*** (*p* < 0.0001)	1.3	0.43	0.12
	5	7.8 ± 0.9	1.8 ± 0.1	*** (*p* < 0.0001)	6	0.22	0.10
	10	30 ± 3.3	4.2 ± 0.3	*** (*p* < 0.0001)	26	0.14	0.04
*Dabcyl*–Arg_6_–Lys(Cf)–*NH*_*2*_	1	2.5 ± 0.2	0.6 ± 0.04	*** (*p* < 0.0001)	1.9	0.25	0.14
	2.5	24 ± 1.3	1.9 ± 0.3	*** (p < 0.0001)	22	0.08	0.04
	5	59 ± 2.5	6.1 ± 0.8	*** (*p* < 0.0001)	53	0.10	0.05
	10	149 ± 6	18 ± 2	*** (*p* < 0.0001)	131	0.12	0.04
*Cf*–Arg_9_	1	2.9 ± 1.3	0.6 ± 0.1	NS (*p* = 0.186)	–	–	0.06
	2.5	20 ± 3.5	1.6 ± 0.2	** (*p* = 0.0033)	18	0.08	0.03
	5	34 ± 4	4.3 ± 0.5	*** (*p* = 0.0009)	30	0.12	0.07
	10	52 ± 6	10 ± 2	** (*p* = 0.0014)	42	0.20	0.09

^a^Cells were incubated with *Dabcyl*–Arg_4_–Lys(*Cf*), *Dabcyl*–Arg_6_–Lys(*Cf*) and *Cf*-Arg_9_ for 60 min. Cell lysis was performed to allow enzymatic cleavage of all the intracellular peptide and to access to total peptide internalisation. At 37 °C, these data reflect endocytosis and translocation; at 4 °C these data reflect translocation only. Statistical analysis with unpaired *T*-test was done to evaluate difference significancy between internalisation mean values measured at 37 °C and 4 °C

These results indicate clearly that tetraarginine shows direct penetration at lower μM concentrations and that endocytic uptake requires higher concentration. In contrast, hexaarginine internalisation happens mainly via endocytosis. This is in accordance with the observations by confocal microscopy. Although the nature of the two peptides (tetra- and hexaarginine) are very similar (highly positively charged peptides) the results indicate that they are different in the mechanism of their internalisation or the interaction with cell-surface components involved in one or the other internalisation pathways. Hexaarginine needs higher concentration for direct penetration (the threshold concentration is between *c* = 5–10 µM), while tetraarginine can go through the cell membrane directly at low concentration and it can induce endocytosis only at higher concentration (starting between 5 and 10 µM). It is worth mentioning in all cases that decreasing temperature to 4 °C can also inhibit translocation (direct passage from the extracellular compartment to the cytosol of cells by membrane perturbation), because membrane fluidity is also negatively impacted. Thus, the translocation pathway measured is likely underestimated herein.

We have then compared the values measured by fluorometry at 37 °C and 4 °C for one given peptide and found that these values are significantly different. In this latter case, we could calculate the difference between internalisation values at the two temperatures (Table 2), which is likely reflecting the contribution of endocytosis in the uptake process. Interestingly, up to 5 μM concentration tetraarginine internalises mainly by translocation compared to hexaarginine and nonaarginine (Table 2: 4 °C/37 °C internalisation ratios obtained by fluorometry). In contrast, hexaargininine and nonaarginine mainly internalise by endocytosis. Strikingly, *Dabcyl*–Arg_6_–Lys(*Cf*)–*NH*_*2*_ is more efficiently internalised than *Cf*–Arg_9_. This result highlights that the number of positive charges is not a crucial parameter for high internalisation efficiency of peptides as already reported (Bechara et al. 2013). This observation further supports the important role of internalisation enhancer of the Dabcyl moiety when incorporated in cell-penetrating peptides. Finally, as expected the 4 °C/37 °C internalisation ratios obtained by flow cytometry show that translocation is indeed not detected in these conditions for tetraarginine, because of quenching of the native peptide. Otherwise the same variation trends in fluorescence are observed for the other two peptides.

### Cytostatic activity of conjugates containing antitumor drug and *Dabcyl*–Arg_6_–Lys–*NH*_*2*_

During the analysis of cellular uptake, the *Dabcyl*–Arg_6_–Lys(*Cf*)–*NH*_*2*_ had highly affected the cell viability. Thus, cytostatic effect of tetra- and hexaarginine derivatives was measured on HL-60 cells. The *Dabcyl*–Arg_6_–Lys(*Cf*)–*NH*_*2*_ had lower IC_50_ value (21.8 µM) than that of *Dabcyl*–Arg_4_–Lys(*Cf*)–*NH*_*2*_ (52.5 μM; Table 3). While the *Dabcyl*–Arg_6_–Lys–*NH*_*2*_ had no cytostatic effect up to *c* = 100 μM. As the Dabcyl labelled hexaarginine and Cf alone do not have any cytostatic effect on cells, there should be some interaction in their conjugates resulting in increased toxicity. The toxicity of our construct was studied on the CHO-K1 cells, too. The cells were treated with the conjugate (*Dabcyl*–Arg_6_–Lys(*Cf*)–*NH*_*2*_) using different concentrations for 1 h. The conjugate has no cytotoxic effect under this condition (Supplementary information). Based on results, our new Dabcyl modified hexaarginine might be able to deliver efficiently cargo into cells even at low micromolar concentration range. To verify this idea, some conjugates containing antitumor drug were synthesised. In these conjugates daunomycin (Dau) or pentaglutamylated methotrexate (MTX) was attached to the *Dabcyl*–Arg_6_–Lys–*NH*_*2*_ peptide. In case of Dau-conjugates the amino group of Dau was first succinylated and the carboxyl group of this derivative was reacted with the ε-amino group of lysine (*Dabcyl*–Arg_6_–Lys(*Suc*–*Dau*)–*NH*_*2*_).

**Table 3:** Cytostatic activity of conjugates on HL-60, MCF-7 and MDA-MB-231 cell lines

Conjugate	IC_50_ (μM)^a^
	HL-60
*Dabcyl*–Arg_4_–Lys(*Cf*)–*NH*_*2*_	52.5 $\pm 1$ 2.3
*Dabcyl*–Arg_6_–Lys(*Cf*)–*NH*_*2*_	21.8 $\pm 8.2$
*Dabcyl*–Arg_6_–Lys–*NH*_*2*_	> 100
*Dabcyl*–Arg_6_–Lys(Suc–Dau)–*NH*_*2*_	16.0 $\pm$ 2.8
Suc–Dau	33.5 $\pm$ 1.1

	MCF-7	MDA-MB-231
*Dabcyl*–Arg_4_–Lys(MTX)–*NH*_*2*_	44.2 $\pm 19$.0	70.9 $\pm 30.1$
*Dabcyl*–Arg_6_–Lys(MTX)–*NH*_*2*_	46.4 $\pm$ 18.5	50.2 $\pm 16.0$
*Dabcyl*–Arg_6_–Lys(Glu_5_–MTX)–*NH*_*2*__1#	71.6 $\pm$ 6.8	14.2 $\pm$ 0.2
*Dabcyl*–Arg_6_–Lys(Glu_5_–MTX)–*NH*_*2*__2#	55.6 $\pm$ 11.6	12.4 $\pm$ 0.2
MTX–Arg_8_	~ 100^b^	> 100^b^
*Dabcyl*–Arg_4_–Lys(Suc–Dau)–*NH*_*2*_	16.5 $\pm 4.5$	14.8 $\pm$ 4.2
*Dabcyl*–Arg_6_–Lys(Suc–Dau)–*NH*_*2*_	11.4 $\pm$ 2.6	13.2 $\pm$ 1.0
Suc-Dau	24.5 $\pm$ 7.1	> 100^b^

^a^The cells were incubated with the compound for 3 h, after cultured in serum-containing medium for 3 days. The IC_50_ values were determined by MTT assay as described in the text. Standard deviation values (sd) are also presented

^b^~ 100 and > 100 mean that the compound has low or no cytostotatic activity at 100 μM

HL-60 human leukemic cells, MCF-7 human breast adenocarcinoma and MDA-MB-231 human triple negative breast adenocarcinoma cells were treated with Dau-containing conjugates. All conjugates were active and resulted in cytostatic effect on all of the cells (Table 3). In case of HL-60 cells the cytostatic activity is comparable with hexa- and octaarginine-containing conjugates (Bánóczi et al. 2008b). The conjugates of tetra- and hexaarginine derivatives showed similar activity on both breast cancer cells (MCF-7 and MDA-MB-231). Although the amount of internalised peptides was significantly different and dependent on the number of arginine residues, they had same antitumor activity on both cell lines. Similar results was noticed when methotrexate was used as antitumor drug. Both peptide-conjugates had almost identical activity on the breast cancer cells independently of the length of peptides. On the sensitive MCF-7 cells the MTX conjugates were slightly more active than on resistant MDA-MB-231 cells. In case of hexaarginine derivative the cytostatic activity of conjugates containing pentaglutamylated MTX was studied on MCF-7 and MDA-MB-231 cells. The last step in the synthesis of MTX containing conjugates was the coupling of MTX. As the MTX has two carboxylic groups (α and γ) it can react with both resulting in two isomer conjugates. In case of the pentaglutamylated derivative the two isomers could be separated during RP–HPLC purification. We used them without clarifying which conjugates containing the MTX in α or γ peptide bond. It was noticed that the mode of coupling did not change the bioactivity of conjugates, because both had very similar effect on both cell lines (Table 3). The two breast cancer cell lines differ to each other only in their receptor status (Subik et al. 2010). Because of this difference their response to various drugs may significantly dissimilar. MCF-7 cells are sensitive to MTX and Suc–Dau treatment, while MDA-MB-231 cells are essentially MTX and Suc–Dau resistant. In our previous work we compared the effectiveness of MTX conjugates of penetratin and octaarginine (Szabó et al. 2016). We noticed that the presence of pentaglutamyl moiety decreased dramatically the cellular uptake of both cell-penetrating peptide and only the penetratin conjugates showed activity. Almost all of the octaarginine conjugates studied were ineffective. Using Dabcyl modified tetra- and hexaarginine, the conjugates had activity on both cell lines. Interestingly the activity of MTX containing conjugates was higher on sensitive MCF-7 cells while the pentaglutamylated derivatives’ activity was higher on MTX resistant MDA-MB-231. These results suggest that *Dabcyl*–Arg_6_–Lys–*NH*_*2*_ can be considered as CPP for successful delivery of negatively charged cargo, which cannot do efficiently by octaarginine.

## Conclusion

**Scheme 1:**
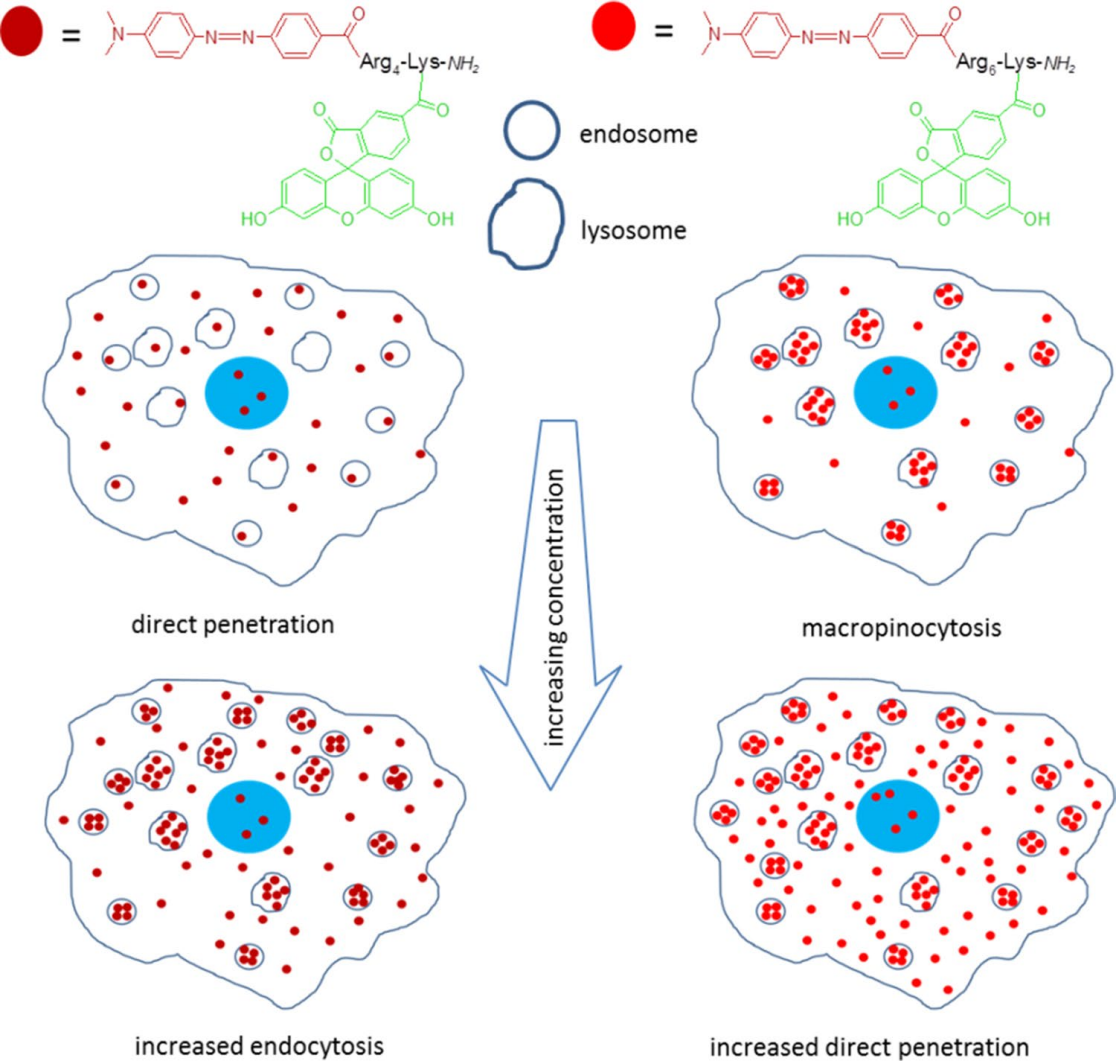
Schematic diagram of the main pathways of penetration. The internalisation pathways of the two peptides were dependent on the length of peptide and the applied concentration

Cell-penetrating peptides are promising tools to deliver biological active compounds into cells. Although they were applied successfully in many cases, there are some limitations. The mechanism of the internalisation often hampers their applicability, because of the endocytosis caused encapsulation inside the cells. Here we showed that modification of short oligoarginines (tetra- and hexarginine) with Dabcyl group not only enhances the internalisation, but push its mechanism towards the direct translocation (Scheme 1). Although these short oligoarginines are very similar; they are highly positively charged and disordered in physiological conditions; the effect of Dabcyl group was different and dependent on the number of Arg residues. Though it increased the cellular uptake of both peptides, the effect was more pronounced in case of hexaarginine. At the same time it decreased the threshold concentration of direct translocation of hexaraginine, which may be very important in the efficacy of drug delivery. In case of tetraarginine, the presence of Dabcyl group it not only enhanced the internalisation of this short peptide, but shifted the internalisation mechanism to direct translocation at low concentration range and thus resulted in a threshold concentration of the endocytosis. The conjugates containing antitumor drug presented the applicability of this derivative as delivery agent.

## Supplementary Information

Below is the link to the electronic supplementary material.

Supplementary file1 Supporting Information Analytical methods, analytical HPLC chromatograms and ESI-MS spectrum of conjugates, in vitro cytotoxicity data. (DOCX 379 kb)

## Data Availability

All the data supporting the results of this study are available within the article and its supplementary materials.
