# Supplementary material for: Influence of the Dabcyl group on the cellular uptake of cationic peptides: short oligoarginines as efficient cell-penetrating peptides
Source: Amino Acids. 2021 May 25;53(7):1033–49. doi: 10.1007/s00726-021-03003-w (PMC8241751; doi:10.1007/s00726-021-03003-w)
Supplement: Supplementary file 1 — Supplementary file1 Supporting Information Analytical methods, analytical HPLC chromatograms and ESI-MS spectrum of conjugates, in vitro cytotoxicity data. (DOCX 379 kb) [file 726_2021_3003_MOESM1_ESM.docx]

Supporting Information

**Increased cellular-uptake by modification with Dabcyl group - short oligoarginines as efficient cell-penetrating peptides**

Ildikó Szabó^1^, Françoise Illien^2^, Levente E. Dókus^1^, Mo’ath Yousef^3^, Zsuzsa Baranyai^1^, Szilvia Bősze^1^, Shoko Ise^4^, Kenichi Kawano^4^, Sandrine Sagan^2^, Shiroh Futaki^4^, Ferenc Hudecz^1,3^, Zoltán Bánóczi^3^

*^1^* *ELKH-ELTE Research Group of Peptide Chemistry, Eötvös L. University, Budapest, Hungary*

*^2^Sorbonne Université, École normale supérieure, PSL University, CNRS, Laboratoire des biomolécules, LBM, 75005 Paris, France.*

*^3^Department of Organic Chemistry, Eötvös L. University, Budapest, Hungary*

*^4^Institute for Chemical Research, Kyoto University, Uji, Kyoto 611-0011, Japan*

*E-mail: zoltan.banoczi@ttk.elte.hu*

**1. Characterisation of compounds by analitical RP-HPLC – System 1** Characterisation with Analytical RP-HPLC was performed on Zorbax SB C18 column (150_4.6mm I.D.) with 5 µm silica (100 Å pore size) column. Linear gradient elution was used: 0 min 0% B; 2 min 0% B; 22 min 90% B with eluent A (0.1% TFA in water) and eluent B (0.1% TFA in acetonitrile-water (80:20, v/v)), flow rate: 1 mL/min, ambient temperature. Peaks were detected at λ = 220 nm.


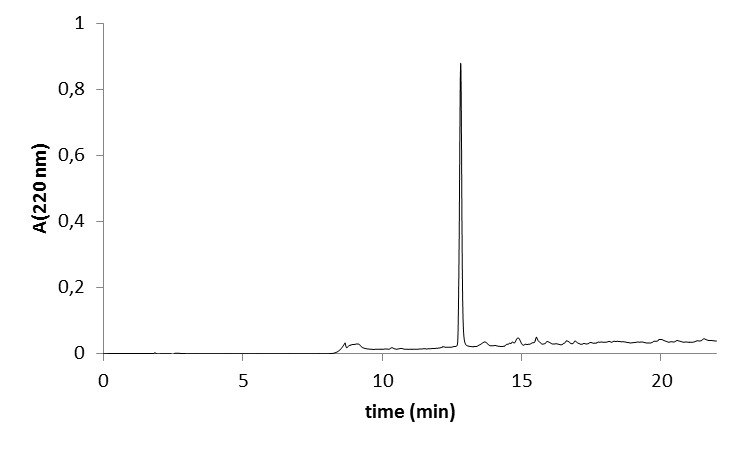


*Ac*-Arg_4_-Lys(Cf)-*NH_2_*


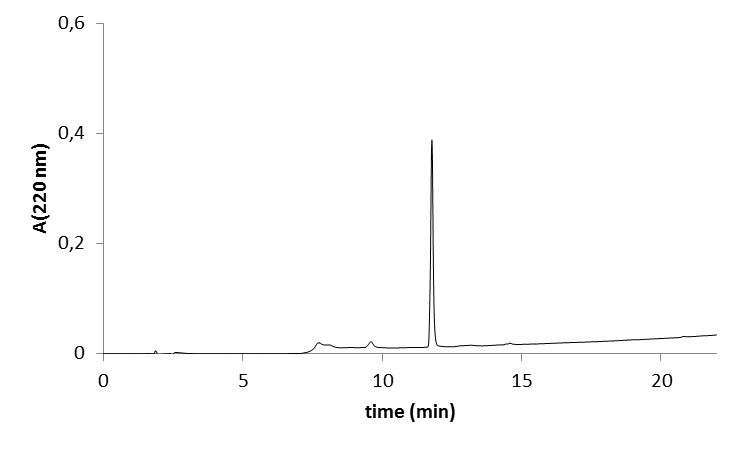


*Ac*-Arg_6_-Lys(*Cf*)-*NH_2_*


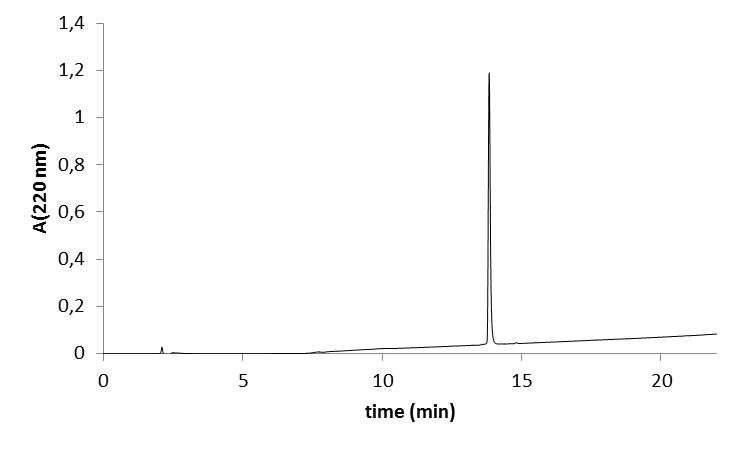


*Dabcyl*-Arg_4_-Lys(*Cf*)-*NH_2_*


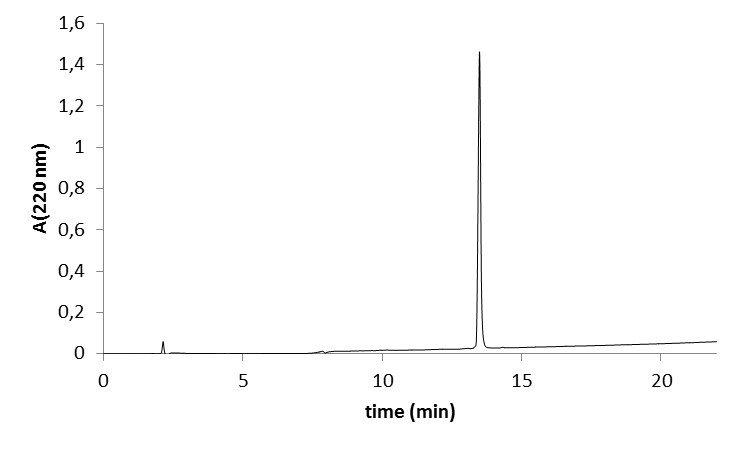


*Dabcyl*-Arg_6_-Lys(*Cf*)-*NH_2_*


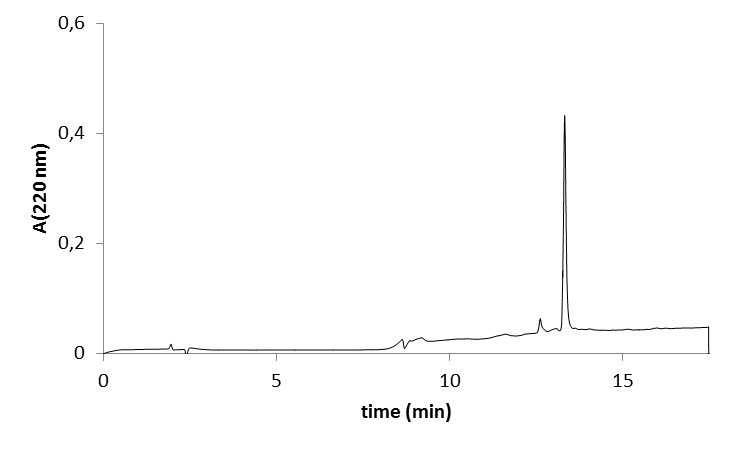


*Ac*-Arg_6_-Lys(*Rh*)-*NH_2_*


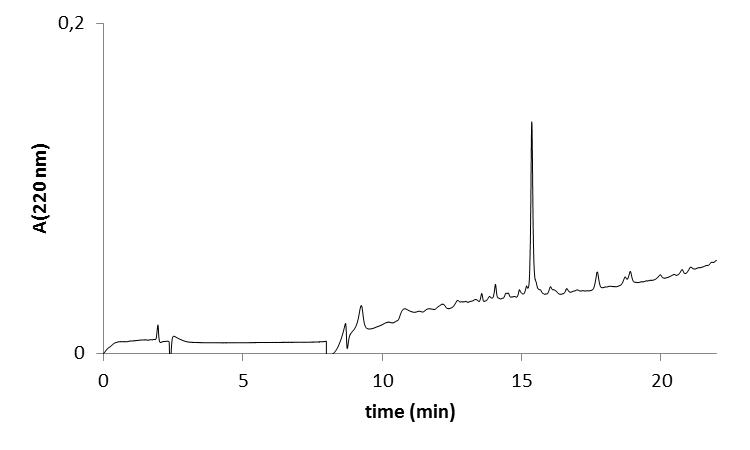


*Dabcyl*-Arg_6_-Lys(*Rh*)-*NH_2_*


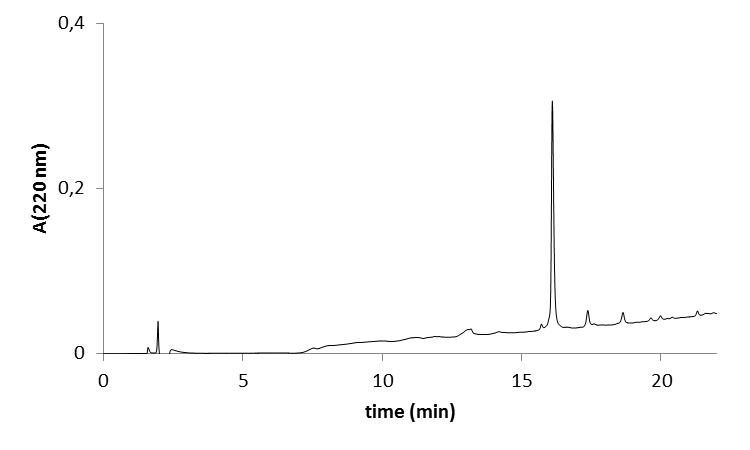


*Dabcyl*-Arg_6_-Lys(*Suc-Dau*)-*NH_2_*

**2. Characterisation of compounds by analitical RP-HPLC – System 2** Analytical RP-HPLC was performed on a Knauer (Herbert Knauer GmbH, Berlin, Germany) HPLC system using a Phenomenex Jupiter C18 column (250x4.6mm I.D.) with 5 µm silica (300 Å pore size) (Torrance, CA USA) as a stationary phase. Linear gradient elution (0 min 0% B; 5 min 0% B; 50 min 90% B) with eluent A (0.1% TFA in water) and eluent B (0.1% TFA in acetonitrile-water (80:20, V/V)) was used at a flow rate of 1 mL/min at ambient temperature. Peaks were detected at λ = 220 nm.


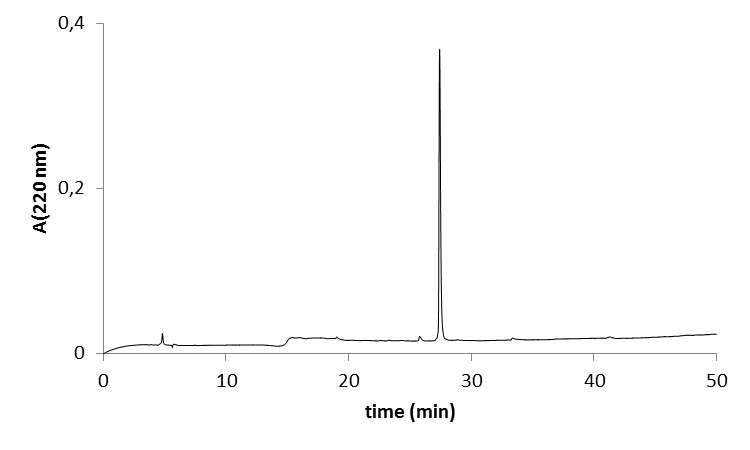


*Dabcyl*-Arg_6_-Lys(*Glu_5_-MTX*)-*NH_2_* 1#


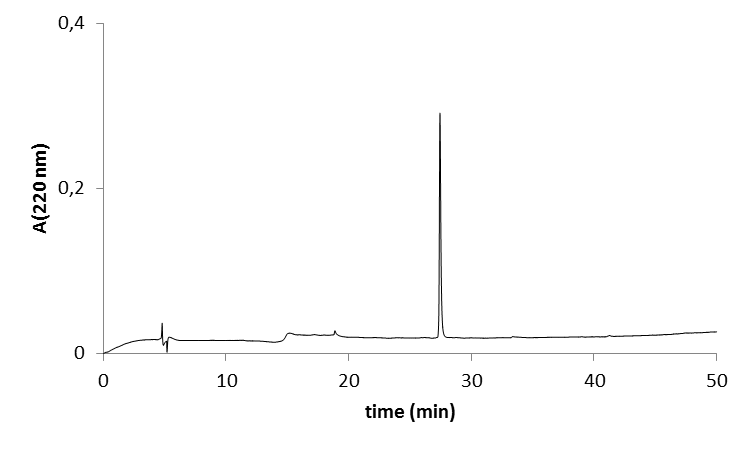


*Dabcyl*-Arg_6_-Lys(*Glu_5_-MTX*)-*NH_2_* 2#

**3. Characterisation of compounds by analitical RP-HPLC – System 3** Characterisation with Analytical RP-HPLC was performed on Jupiter SB C18 column (150_4.6mm I.D.) with 3 µm silica (100 Å pore size) column. Linear gradient elution was used: 0 min 0% B; 2 min 0% B; 22 min 90% B with eluent A (0.1% TFA in water) and eluent B (0.1% TFA in acetonitrile-water (80:20, v/v)), flow rate: 1 mL/min, ambient temperature. Peaks were detected at λ = 220 nm.

**
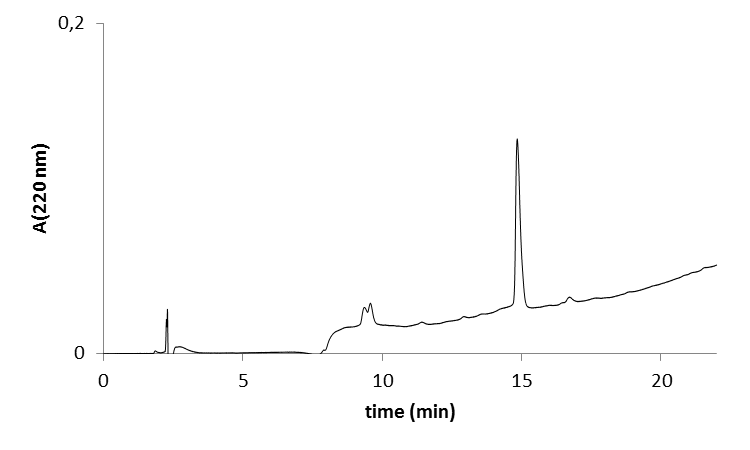
**

*Dabcyl*-Arg_4_-Lys(*Suc-Dau*)-*NH_2_*


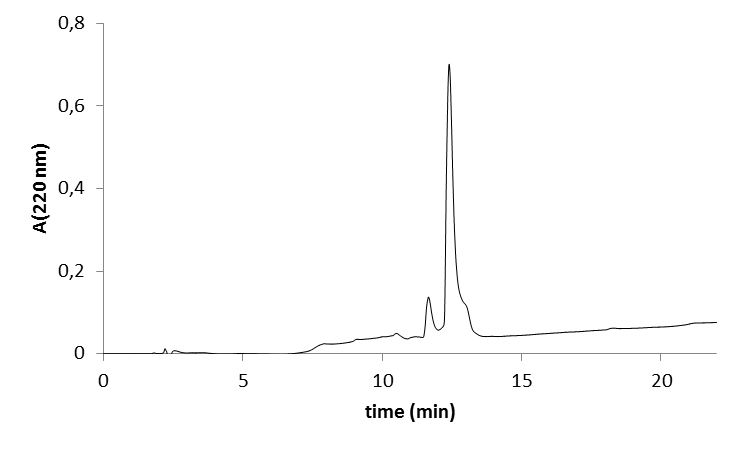


*Dabcyl*-Arg_4_-Lys(*MTX*)-*NH_2_*


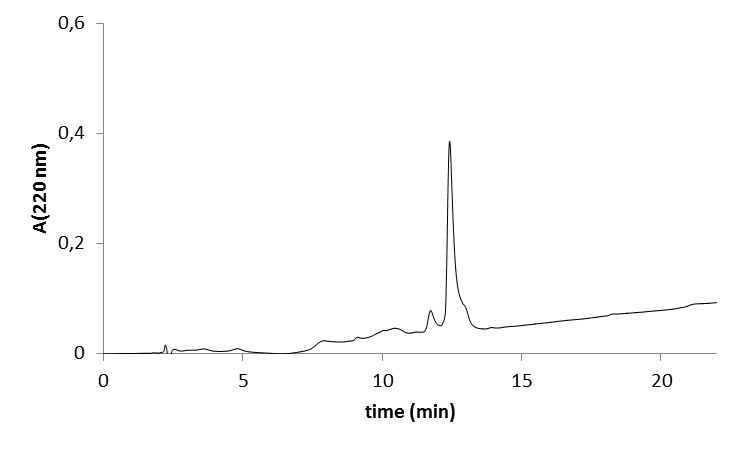


*Dabcyl*-Arg_6_-Lys(*MTX*)-*NH_2_*

**3. Characterisation of compounds by ESI-MS**

The molecular mass of the components as well as the conjugates was determined by ESI-MS. The mass spectrometric analysis was performed on a Bruker Daltonics Esquire 3000 plus (Germany). The samples were dissolved in acetonitrile–water (50:50, V/V), containing 0.1% acetic acid.


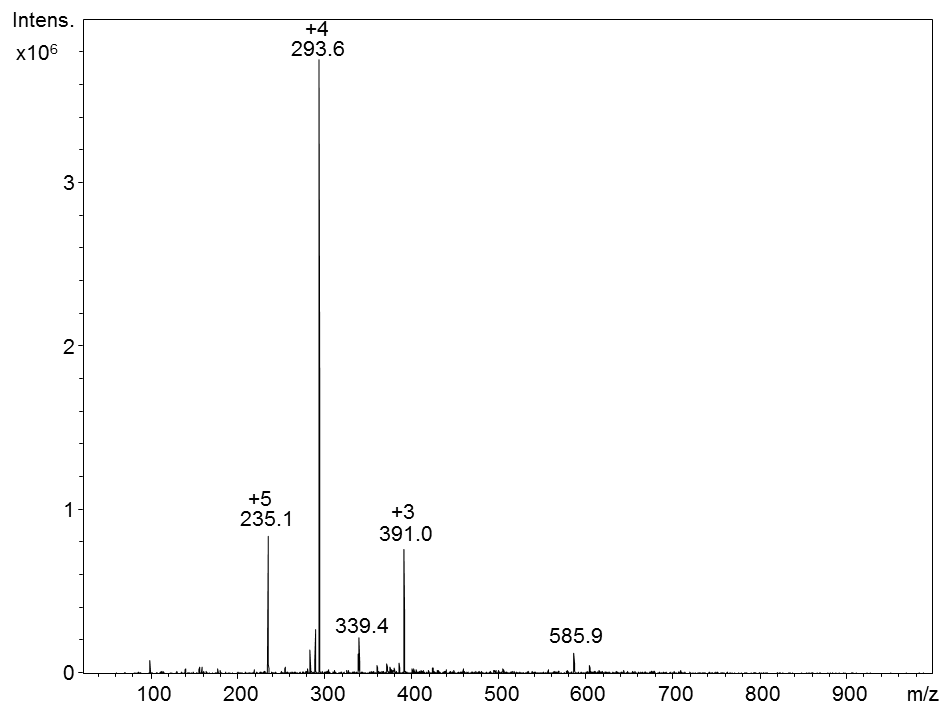


*Ac*-Arg_4_-Lys(*Cf*)-*NH_2_*


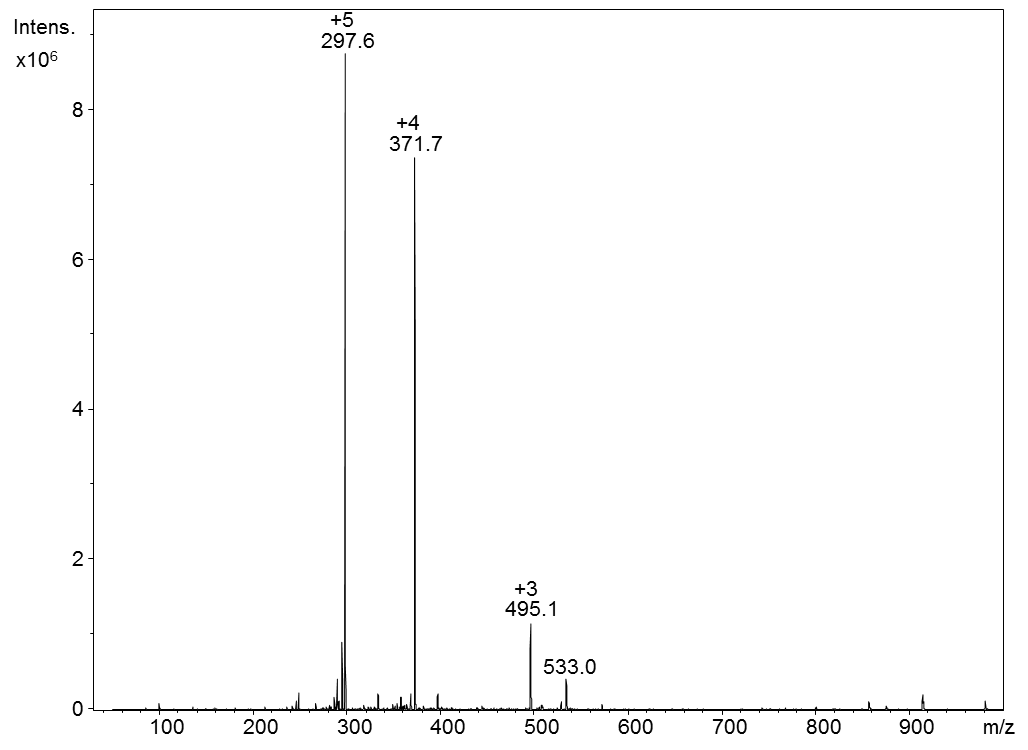


*Ac*-Arg_6_-Lys(*Cf*)-*NH_2_*


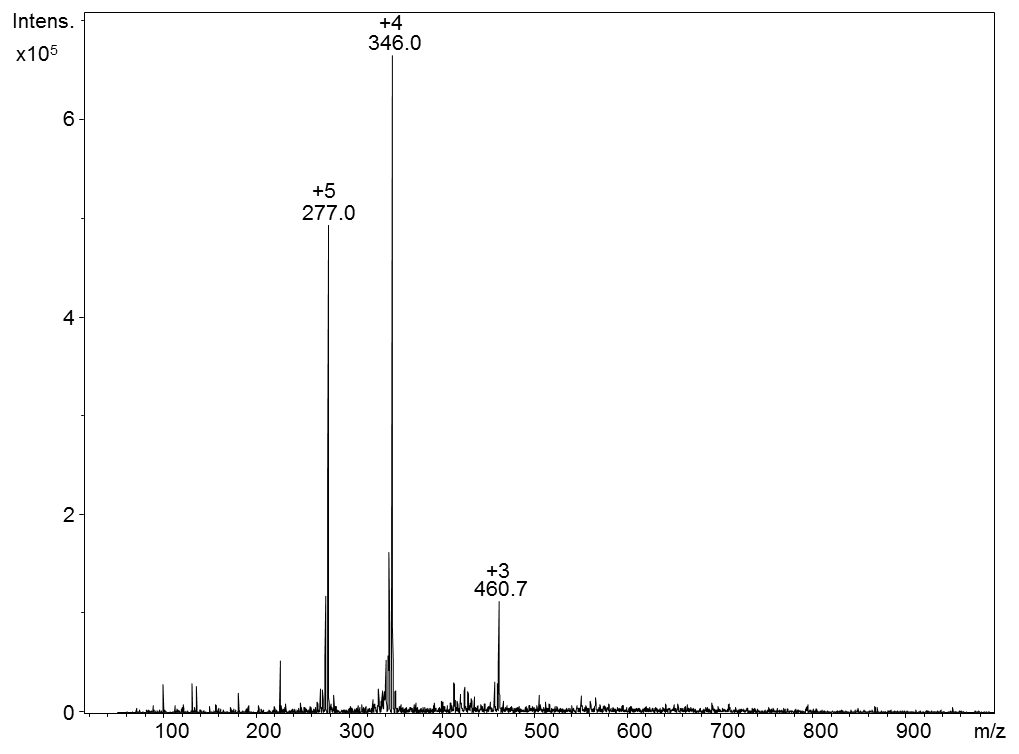


*Dabcyl*-Arg_4_-Lys(*Cf*)-*NH_2_*


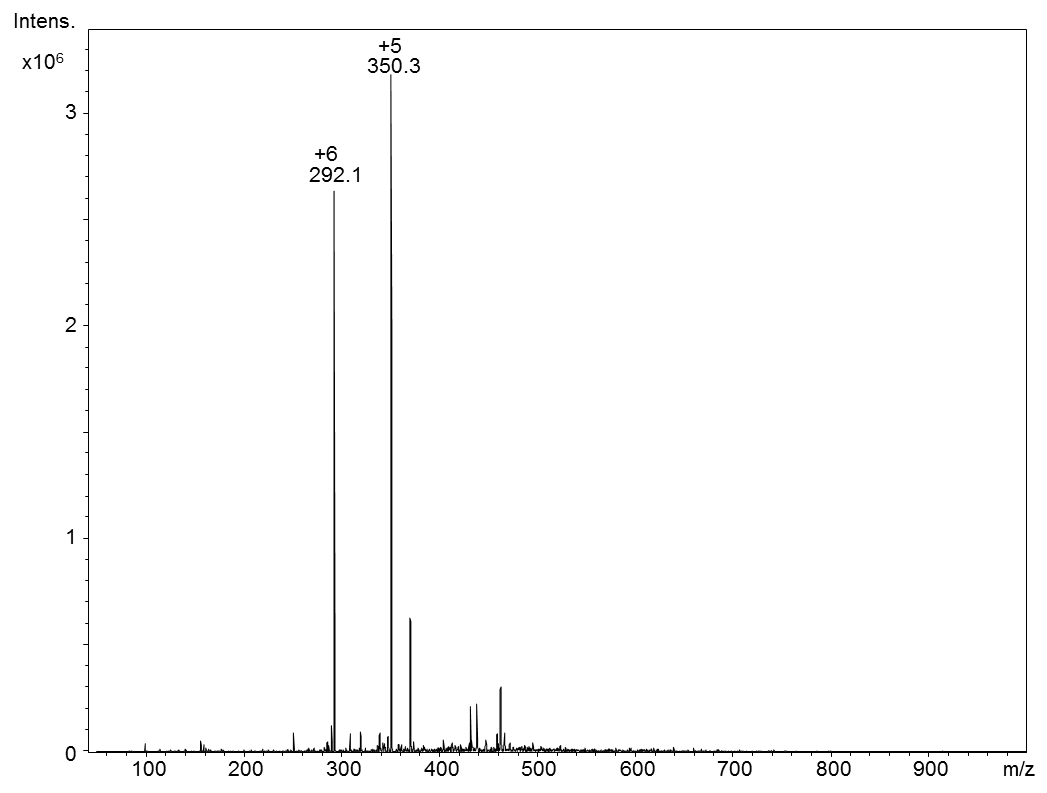


*Dabcyl*-Arg_6_-Lys(*Rh*)-*NH_2_*


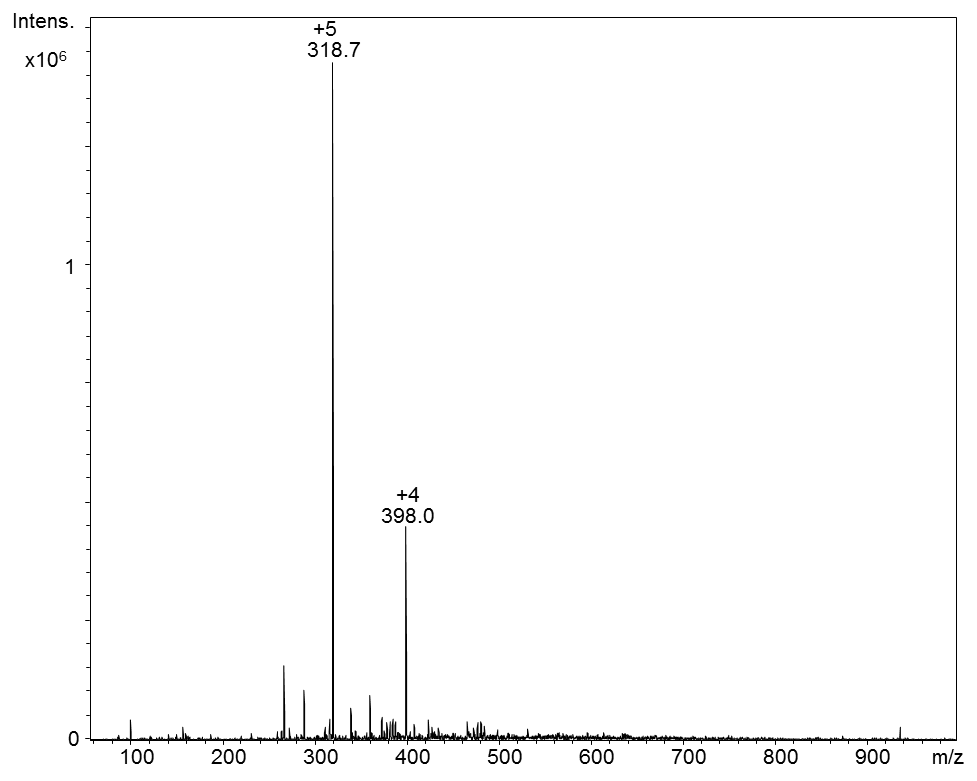


*Dmab*-Arg_6_-Lys(*Cf*)-*NH_2_*


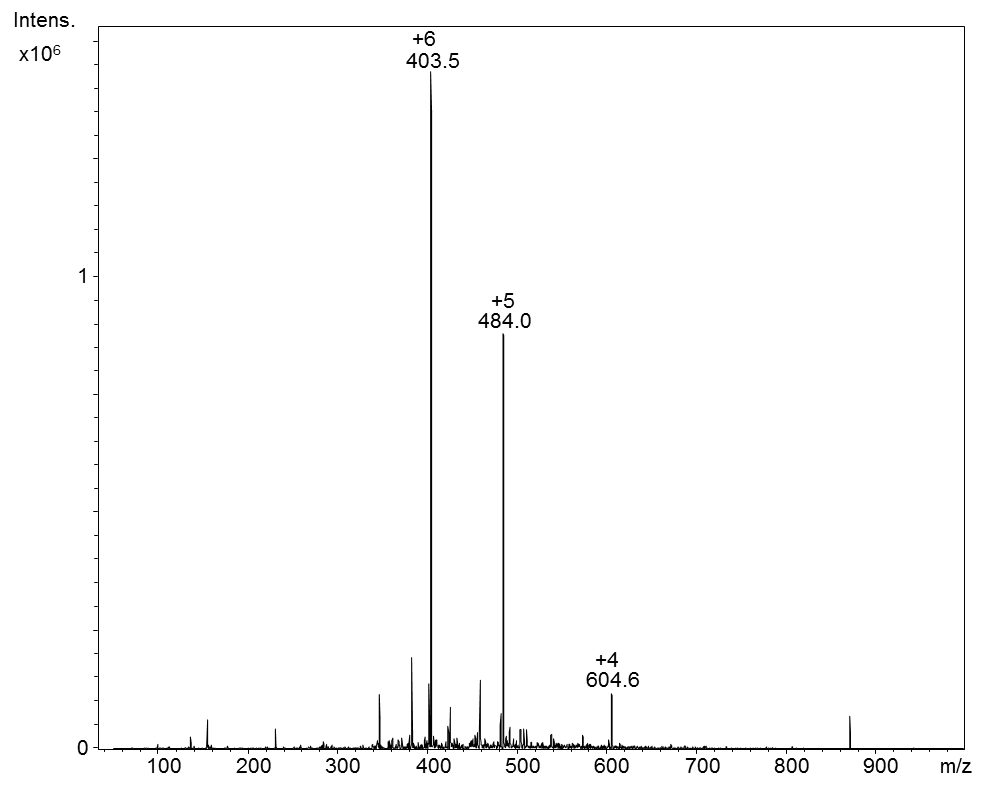


*Dabcyl*-RRRRRRK(*Glu_5_-MTX*)-*NH_2_*_1#


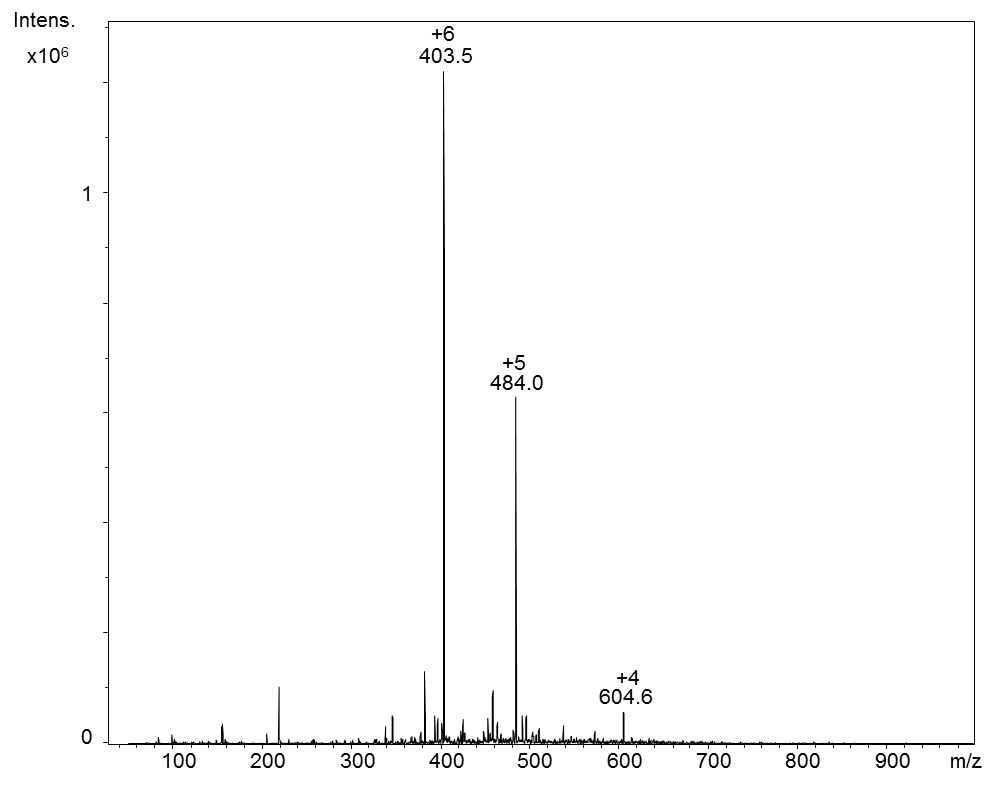


*Dabcyl*-RRRRRRK(*Glu_5_-MTX*)-*NH_2_*_2#


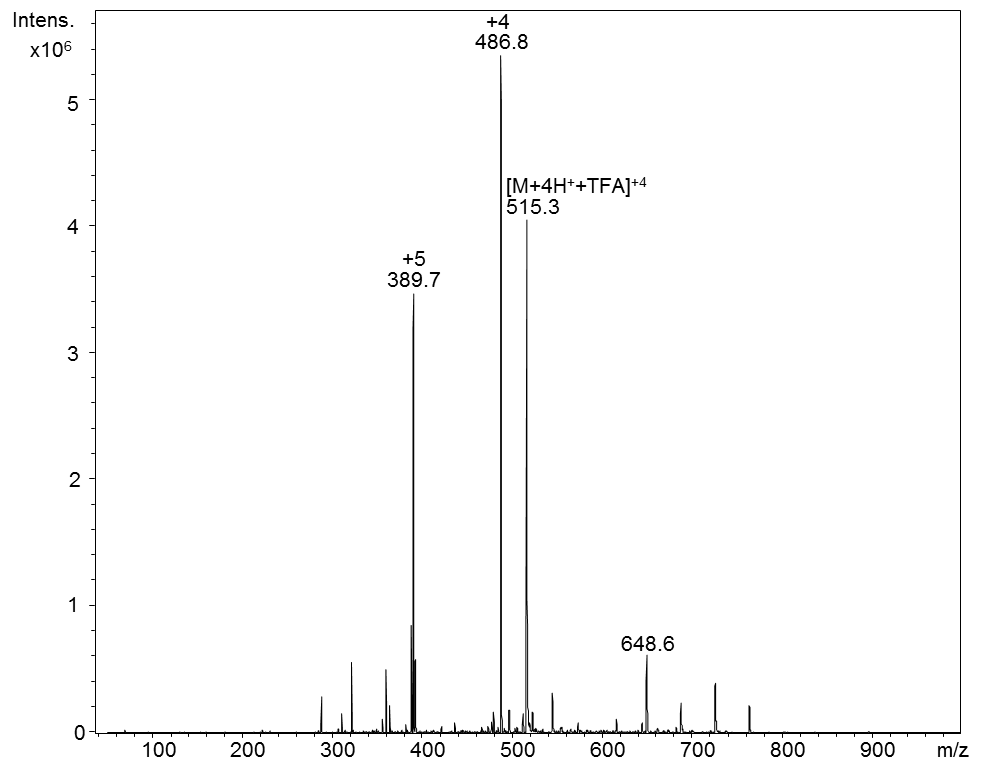


*Dabcyl*-RRRRRRK(*Suc-Dau*)-*NH_2_*


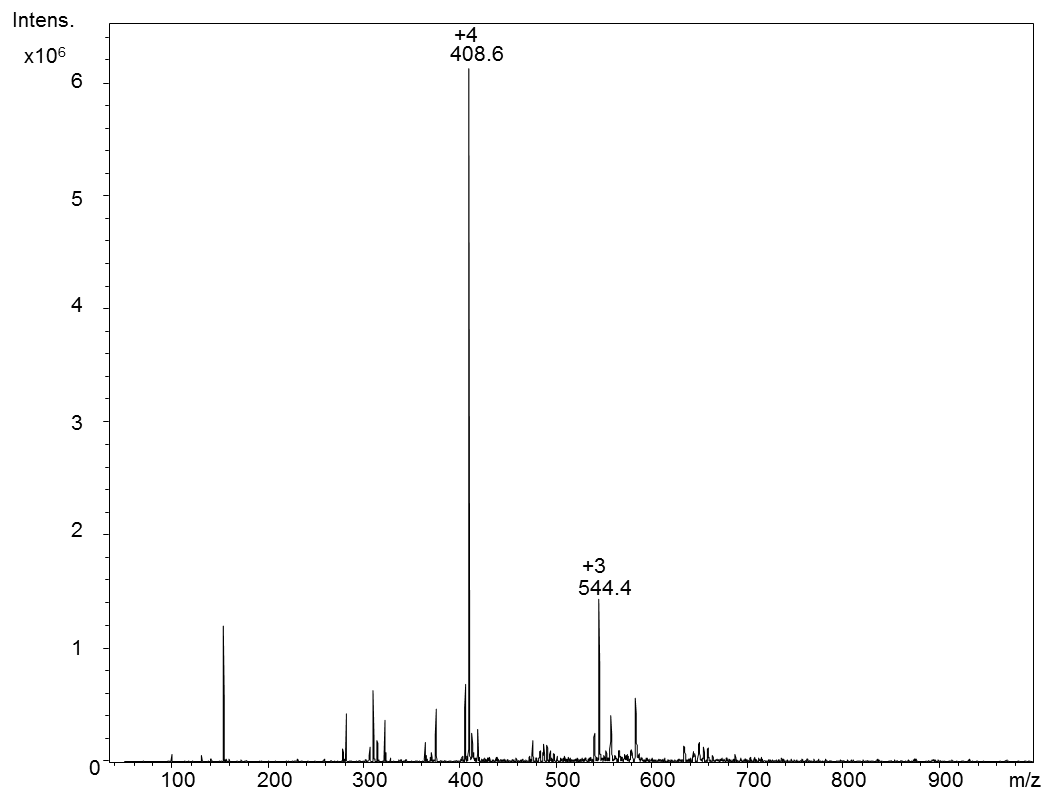


*Dabcyl*-RRRRK(*Suc-Dau*)-*NH_2_*


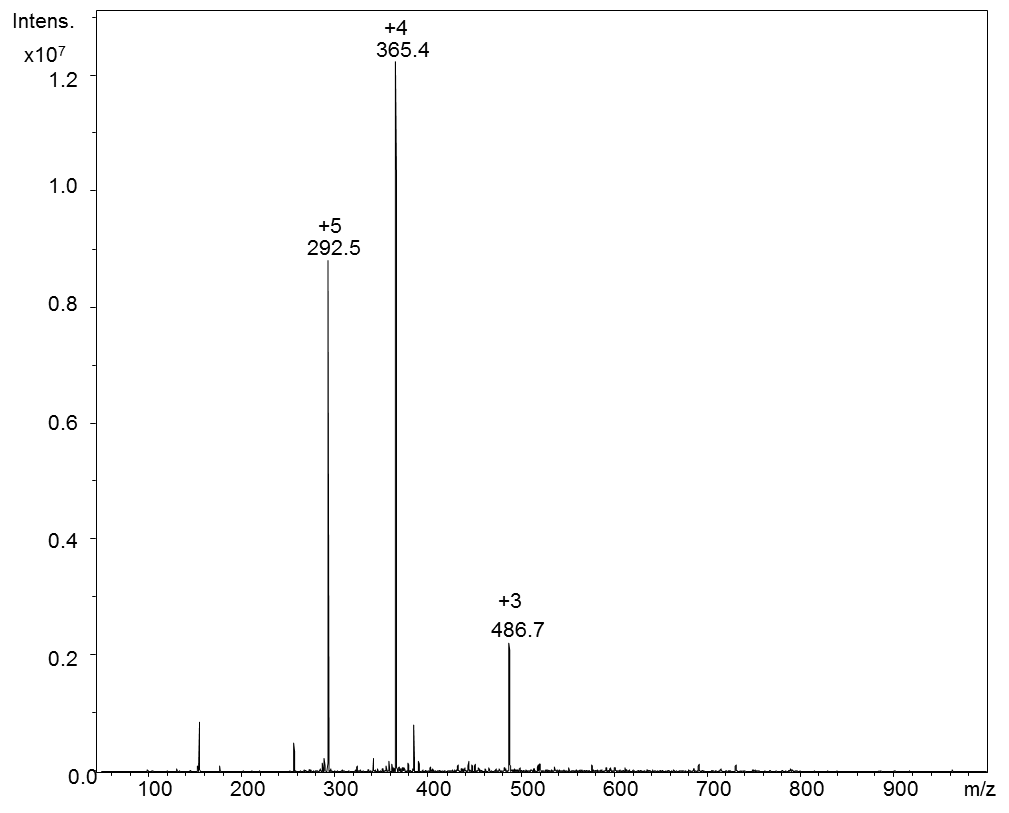


*Dabcyl*-RRRRK(*MTX*)-*NH_2_*


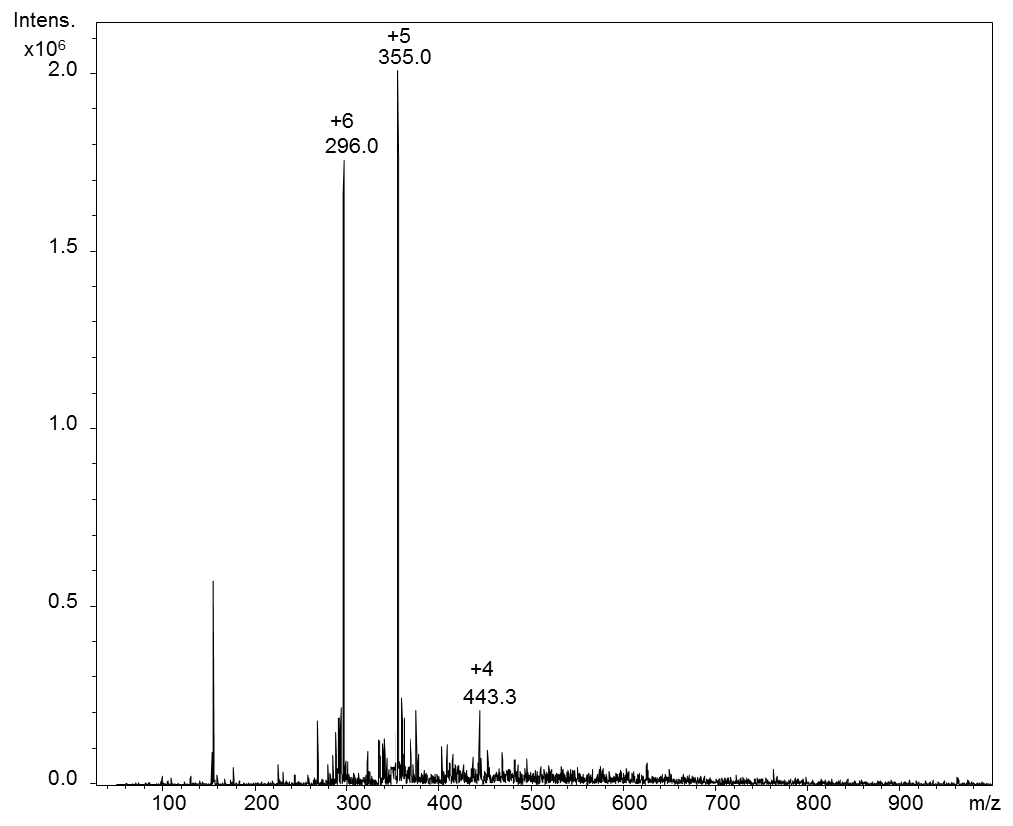


*Dabcyl*-RRRRRRK(*MTX*)-*NH_2_*

**4. *In vitro* cytotoxicity assay**

Cytotoxicity was determined with the Cell Counting Kit 8 (CCK-8) from Dojindo Molecular Technologies using CHO-K1 cells. This colorimetric assay allows to measure the viability of cells. 96-well plates were inoculated with 100 μL/well of a suspension of cells (2 × 10^4^ cells/well). After 24 h of incubation (37°C, 5% CO_2_) different peptide concentrations of *Dabcyl*-Arg_6_-Lys(*Cf*)-*NH_2_* , *Dabcyl*-Arg_4_-Lys(*Cf*)-*NH_2_* and *Cf*-Arg_9_ (0, 1, 2.5, 5, 10 and 20 µM final in DMEM F12) were added and the plate was further incubated for 60 min at 37°C. After washing, the cells were incubated with 100 μL of 10% CCK-8 in DMEM for 3 h and the absorbance was measured at λ= 450 nm with a microplate reader (Polarstar Optima). Controls corresponded to untreated cells with DMEM (negative control, 100% viability) and cells treated with 0.1% of Triton X-100 (positive control, 0% viability).

**Table 1.** Cytotoxicity assays in CHO-K1 cells after 1hr incubation with peptides at different concentrations.
